# Interventions to support parents, families and caregivers in caring for preterm or low birth weight infants at home: A systematic review and meta-analysis

**DOI:** 10.1371/journal.pgph.0005690

**Published:** 2026-02-10

**Authors:** Amie Wilson, Carol Bedwell, Valentina Actis Danna, Natalie Tate, Kerry Dwan, Anayda Portela, Tina Lavender

**Affiliations:** 1 Liverpool School of Tropical Medicine, Liverpool, United Kingdom; 2 University of York, York, United Kingdom; 3 World Health Organization, Geneva, Switzerland; Qatar University College of Medicine, QATAR

## Abstract

The aim of this study was to determine what interventions, approaches, or strategies to support mothers/fathers/caregivers and families in caring for preterm (<37 gestational weeks) or low birthweight (<2,500g) infants in the home have been effective in improving outcomes. We conducted a systematic review and meta-analysis. A comprehensive search of relevant electronic databases, including MEDLINE, Embase, CINAHL and Cochrane Central Register of Controlled Trials was completed in September 2024. Studies were included if they utilised interventions which focused on providing support to participants (mother/father/parents, families or caregivers) to care for their infants in the home. Two reviewers independently screened papers in Covidence and extracted data. Random effects meta-analyses were undertaken. Quality of studies and certainty of evidence were assessed using CASP and GRADE, respectively. Critical outcomes based on WHO preterm and low birthweight criteria comprised infant mortality, morbidity, growth and neurodevelopment. Priority outcomes comprised breastfeeding, care seeking, parent-infant interaction, mother-child attachment and parental health and wellbeing. Forty-seven studies were included. There is some evidence that support interventions may improve outcomes related to infant mortality, improvements in infant growth, exclusive breastfeeding, infant cognitive development, immunisation uptake, and reduction in maternal stress and depression. However, the overall certainty of evidence is low or very low in the majority of studies. We conclude that interventions providing support for parents to care for infants in the home may improve outcomes for this population. There is a need for well-considered large scale support interventions, prioritised and developed with women and families.

## Introduction

In 2019, 2.4 million children died within 28 days of birth with a further estimated 1.5 million deaths in the first year of life [1], the majority of these occurring in Low- and Middle-Income Countries (LMIC)s. Infants who are born prematurely (below 37 weeks gestation) or are of low birth weight (LBW) (<2500g) are at greater risk of mortality and morbidity, with a 2- to 10-fold higher risk of death when compared to babies that are not preterm or of low birthweight [2]. It is estimated that 60–80% of newborns who die are either premature, small for gestational age or premature and small for gestational age [2]. Of those that do survive, associated long term morbidity can impact on their wellbeing decades later [3]. Many advances in neonatal care have led to a reduction in infant mortality. The mortality rate for neonates has reduced from 31 per 1000 live births in 2000–18 per 1000 live births in 2018, but this is still short of the Sustainable Development Goal (SDG) 3 target of 12 per 1000 live births by 2030, and the global aim to end preventable deaths [1]. Whilst the majority of newborns that die, do so in the first month of life [1], a considerable number (1.4m) die during infancy; between 28 days to one year of age, many after facility discharge. Hence, effective interventions to improve infant health and wellbeing in the short and longer term are important in reducing mortality and morbidity.

Parents are the main carers for infants following their discharge from healthcare facilities, yet often feel ill equipped for the task of caring for a preterm or LBW infant at home [4,5]. This can be due to a lack of support, confidence, education or practical and environmental factors [6]. This in turn, can have an impact on parental wellbeing and their subsequent ability to care for their newborn. Evidence suggests that in addition to the stress of caring for a preterm or LBW infant, women are up to 40% more likely to suffer from depression than mothers of healthy infants [7]. Furthermore, in some settings, stigma and community prejudice towards LBW babies may also impact on women’s ability to care for their infant without support [8]. Many reported interventions are conducted within the facility setting, however, the period of time after discharge from health system care may be the most vital in terms of parental needs. It is at this point that parents report feeling overwhelmed and in need of greater support [9]. Psychosocial, emotional and financial support, along with greater understanding of developmental expectations, have been raised as concerns by parents following discharge [6].

Interventions that support parents in caring for their newborns may help improve specific maternal and infant outcomes. A systematic review focused on healthy term infants suggested that nurturing parenting interventions can improve developmental outcomes [10]. A further systematic review demonstrated parent-to-parent support in facility neonatal care increased perceptions of support, reduced maternal stress, and increased mothers confidence in the ability to care for their baby, but had no impact on parenting practices nor on breastfeeding [11]. This review will focus specifically on interventions that aim to support parents to care for their newborn preterm or LBW infant in the home.

## Methods

The systematic review followed standard systematic review principles and is reported in line with PRISMA reporting requirements [12]. The review is registered in the International Prospective Register of Systematic Reviews (https://www.crd.york.ac.uk/PROSPERO/view/CRD42021275525) The review was expanded after registration, from interventions taking place in the home only, to those which were initiated in the facility or in the home, but which related to outcomes measured following discharge. This change was to ensure we captured the effects of facility-based interventions that impacted on parental support following discharge.

### Eligibility criteria

Studies were included if they met the following inclusion criteria (these criteria were applied to all studies):

- Participants were mother/father/parents, families or caregivers of preterm (defined as born before 37 completed weeks gestation) or LBW (defined as a birthweight of less than 2500 grams) infants.
- Included interventions which focused on providing support to participants to care for their infants in the home; including, education and counselling, home visits, family and community mobilisation approaches, and commenced either during pregnancy, before discharge or after discharge/after birth, with outcomes measured in the home.
- Studies which measured critical and priority infant and maternal outcomes (as per the WHO recommendations for care of the preterm or low-birth-weight infant), and which were measured in the home up to 12 months of age. Critical outcomes as per WHO recommendations comprised infant mortality (number of deaths), morbidity (as defined by authors), growth (length or weight) and neurodevelopment (cognitive, motor, measured by a validated scale). Priority outcomes as per WHO recommendations comprised breastfeeding (exclusive and/or duration), care seeking (hospital visits or contact with health services), parent-infant interaction, mother-child attachment (measured by a validated scale) and parental health and wellbeing (stress, anxiety depression, quality of life measured by a validated scale). Critical and priority outcomes were based on WHO criteria [13] and following discussion with WHO expert panel.
- Randomised controlled trials or quasi-experimental studies.
- Studies were included regardless of high-, middle- or low-income setting.
- Were published in full text between 2000 and 2024

Studies were excluded if their focus was on clinical care only, did not report data from preterm or LBW infants, did not have a control group or were of qualitative design, or were published before 2000, to ensure studies of current relevance were included.

A comprehensive search of relevant electronic databases included Medical Literature Analysis and Retrieval System Online (MEDLINE), Excerpta Medica Database (Embase), Cumulative Index to Nursing and Allied Health Literature (CINAHL) and Cochrane Central Register of Controlled Trials, using a detailed search strategy (see S1 Text). Citation searching was used to identify any further studies meeting the inclusion criteria. The search was completed between August and September 2024.

### Screening, data extraction and analysis

Search results were managed through the Covidence software platform (https://www.covidence.org/), to allow for removal of duplicates and screening. Two reviewers independently screened all papers on title and abstract and reviewed the remaining full-text papers for inclusion, in accordance with the eligibility criteria. Any discrepancies between the reviewers were resolved during a group meeting to discuss and resolve each conflicted decision and achieve consensus, additional author input was sought if consensus was not achieved.

A data extraction form, tailored to the review parameters was used in conducting data extraction, which was conducted independently and in duplicate. The data extracted was organised into domains related to the intervention type and outcome.

There was considerable clinical and methodological heterogeneity across the studies in terms of study designs, interventions and outcomes. Studies were grouped into intervention type, based on the main characteristics of the intervention. A variety of outcome measures and timepoints were used by the included studies. For consistency, similar final timepoint measurements for each study were included in the analysis (e.g., 2–3 months). Where meta-analysis was possible; data from randomised controlled trials were input into Revman web (revman.cochrane.org) and a random effects model was used. For dichotomous data, risk ratio and 95% confidence intervals were reported. For continuous data, mean difference or standardised mean difference and 95% confidence intervals were reported. Standardised mean difference was reported where different measurement scales were used. Effect size is reported in line with Cohen’s effect sizes; 0.2 small effect, 0.5 moderate effect, 0.8 large effect. Heterogeneity between studies limited the opportunity for meta-analysis, with most outcomes synthesised narratively. Heterogeneity between studies was identified by visual inspection of forest plot, chi squared (p < 0.1), and I squared. Heterogeneity was formally explored if number of included studies permitted, in the absence of this, the reasons for the presence of statistical heterogeneity were explored by examining the characteristics of the studies, populations and the interventions. Non-randomised trials are reported narratively to explore the potential difference in observed effect compared to the randomised evidence.

### Patient and public involvement

There was no direct patient and public involvement in this systematic review.

### Risk of bias and certainty assessment

Quality assessment and risk of bias was completed using CASP [12] by two reviewers, independently. Certainty of the body of evidence was assessed using Grading of Recommendations Assessment Development and Evaluation (GRADE) approach, as outlined in the GRADE Handbook (https://gdt.gradepro.org/app/handbook/handbook.html), for each reported outcome by two reviewers independently. This GRADE approach allows for evidence certainty to be downgraded according to five domains: risk of bias; inconsistency; indirectness; imprecision; and publication bias. The results of the assessment for each study relate to one of four grades: high, moderate, low or very low. Studies and outcomes were included regardless of quality or certainty of evidence given the diversity of interventions, allowing for the range of studies, interventions and outcomes to be reviewed.

Publication bias would have been investigated by constructing a funnel plot if ten or more studies were included in a meta-analysis.

## Results

**Fig 1:**
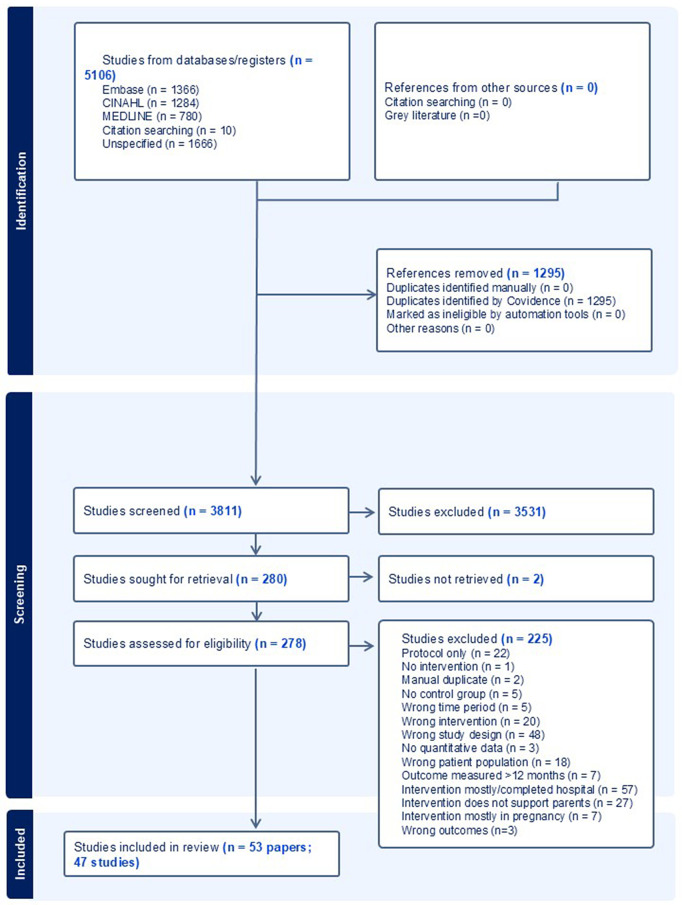
Prisma flowchart.

A total of 5106 papers were identified and screened for inclusion. After removal of duplicates and screening of title and abstract 280 papers received full-text review. A total of 53 papers, related to 47 studies, were included in the final review. See PRISMA flowchart, Fig 1 for details.

### Characteristics of included studies

Thirty-four studies were randomised controlled trials (RCTs), seven pilot RCTs, three quasi-experimental studies, and three before-and-after intervention studies, comprising a total of 12,699 participants, all of whom were mothers (see Table 1 for characteristics of included studies). The majority of studies (n = 29) were based in high-income settings (Australia, Canada, Denmark, Finland, Greece, Hong Kong, Netherlands, Norway, South Korea, Sweden, UK, USA), ten in upper middle-income (China, Taiwan, Jamaica, Turkey, Indonesia), and eight in lower middle-income settings (Bangladesh, Egypt, India, Iran, Philippines). None of the studies were based in low-income settings.

**Table 1:** Study characteristics.

Author Year	Design	Population	Country	Intervention	Control	Outcome	Quality assessment Y = yes; U = unsure; N = no										
1. clearly focused research question?	2. assignments randomised?	3. all participants accounted for?	4. Blinding to intervention a. Participants b. investigators c. outcome assessors c.	5. groups similar at start?	6. study groups treated equally?	7. effects reported comprehensively?	8. Was the precision reported?	9. benefits outweigh harms and costs?	10. results be applied to your local population/ context?	11. intervention provide greater value than any of the existing interventions?
Ahmed 2008 [14]	RCT	Preterm babies below 37 weeks	Egypt	Education and counselling intervention. Individual information giving and skills training in breastfeeding delivered by a researcher over 4 sessions in NICU/1 home, birth to 3 months post-discharge.	Usual care routine of the unit which did not include all of the breastfeeding support, but did include psychological and emotional support after birth.	Exclusive breastfeeding at 2–3 months.	Y	U	Y	UUU	U	Y	U	N	Y	N	U
Beigy 2021 [15]	Quasi-exp	Preterm babies born between 28 – 36 weeks	Iran	Education and support intervention. Knowledge and skills based on the mothers needs, awareness, and skill in caring for their infant during two home visits.	Usual care.	Anxiety (State – trait scale) 7 days after discharge	Y	N	N	NNN	Y	Y	Y	Y	U	U	U
Dusing 2015, 2018 [16,17]	RCT	Preterm babies below 37 weeks	USA	Education and counselling intervention. Individual skills training in infant care and development. Delivered by a trained and experienced NICU Physical therapist at least twice in NICU/ twice weekly at home from discharge, birth to 3 months CA.	Usual care included referral to therapy services in the NICU at the medical team discretion and referral to their local Early Intervention (EI) program. EI was provided in accordance with state implementation guidelines under the United States Individuals with Disabilities Education Improvement Act. Parents were offered referral by NICU staff prior to discharge, during visits to the Neonatal Continuing Care Program, or by the study assessment team if requested by a parent	Motor and cognitive development at 6 months; assessed with Bayley Scales of Infant Development (BSID).	Y	Y	Y	UYY	N	Y	Y	N	U	U	U
Fan 2021 [18]	RCT	Preterm babies between 28–32 weeks	China	Education and counselling intervention. Individual information giving and skills training in infant development. Delivered by a researcher with rehabilitation background over 1 training lecture and 3 workshops in the facility with parents performing EI in the home, for 30 days.	Usual care. Standard post-discharge care was provided to all infants, including feeding guidance, strategies for illness and injuries prevention, bonding with parents, scheduled immunization and available support service if necessary. Advice and booklets dedicated to homecare were delivered to all parents.	Infant weight and length at 120 days follow-up.	Y	Y	Y	UUY	Y	Y	Y	Y	Y	U	U
Finlayson 2020 [19]	RCT	Preterm babies below 30 weeks	Australia	Education and counselling intervention. Individual skills training in infant care and development. Delivered by an experienced physiotherapist/occupational therapist over 5 in NICU/ 5 home, birth to 3 months CA.	Usual care which includes allied health support for very preterm infant infants. Usual care included early development education (e.g., written handouts and developmental care plans).	Cognitive development at 4–6 months; assessed with BSID.	Y	Y	Y	UUU	N	Y	Y	N	U	U	U
Fotiou 2016 [20]	RCT	Preterm babies below 37 weeks	Greece	Education and counselling intervention. Group information and skills training, with additional materials, in self-care including relaxation techniques, progressive muscle relaxation (encouraged to practice these techniques twice daily). Delivered by a Researcher over 5 sessions (90 minutes each) in NICU, parents practiced independently for 3 months after discharge, birth to 3 months CA.	Enhanced care. Parents took part in information-based training courses	Maternal anxiety at 1–2 months; assessed with State Trait Anxiety Inventory (STAI).	Y	Y	Y	NNN	Y	Y	Y	N	U	Y	Y
Glazebrook 2007 [21]	Cluster RCT	Preterm babies below 32 weeks	UK	Education and counselling intervention. Individual information giving and skills training in sensitivity to infant. Delivered by a NICU nurse trained in intervention over weekly one-hour sessions until discharge/ optional to continue until 6 weeks following discharge.	Usual care (no detail provided)	Mother-infant interaction at 3 months; assessed with: Nursing Child Assessment Teaching Scale (NCATS). Maternal stress at 3 months follow-up; assessed with Parenting Stress Index (PSI).	Y	Y	Y	UUY	Y	Y	Y	Y	U	Y	U
Jaywant 2020 [22]	RCT	Preterm babies between 28–36weeks	India	Education and counselling intervention. Individual information giving and skills training in infant care and sensitivity to infant. Delivered by Therapist over Daily session in NICU, for 15 days.	Enhanced care. Control group was given counselling regarding breastfeeding, handling, and positioning of infants.	Maternal stress at 15 days follow-up; assessed with parental stress scale (PSS). Maternal depression at 15 days; assessed with Edinburgh Postnatal Depression Scale (EPDS).	Y	Y	Y	NNN	Y	Y	Y	Y	U	U	U
Kaaresen 2005 [23]	RCT	Low birth weight	Norway	Education and counselling intervention. Individual information giving and skills training in self-care and sensitivity to infant. Delivered by NICU nurse trained to deliver intervention Daily one hour for 7 days pre-discharge/ 4 home visits, 7 day pre- to 90 days post-discharge.	Usual care. Standard protocol for discharge of preterm infants, which includes an examination and offer of training in infant massage from the unit’s physical therapist, a clinical examination including visual and hearing screening, and a discharge consultation with one of the doctors.	Maternal stress at 12 months follow-up; assessed with PSI.	Y	Y	Y	UUY	Y	Y	Y	Y	U	U	U
Melnyk 2001, 2006 [24,25]	RCT	Preterm babies between 26–36 weeks & LBW	USA	Education and counselling intervention. Self-directed materials in infant care, development and sensitivity to infant. Self-directed over 4 phase programme, 2–4 days post-birth to 1-week post-discharge.	Enhanced care. Audiotaped and written information about hospital services, routine discharge information, and education about immunizations across four phases.	Cognitive development at 4–6 months; assessed with BSID. Maternal anxiety at 1–2 months; assessed with State Trait Anxiety Inventory (STAI). Maternal depression at 6 months; assessed with Profile of Mood States.	Y	Y	Y	UUY	Y	Y	Y	N	U	U	U
Milgrom 2013 [26]	RCT	Preterm babies below 30 weeks	Australia	Education and counselling intervention. Individual information giving and skills training in self-care and sensitivity to infant. Delivered by a Psychologist with experience of preterm populations Weekly for 9 weeks in NICU/ at home.	Usual care. Standard best-practice procedures for the care of preterm infants occurred at both NICUs. This included Developmental Care delivered by nursing and medical staff.	Infant temperament at 6 months; assessed by Short Temperament Scale.	Y	Y	Y	UUY	Y	Y	Y	N	U	U	U
Moudi 2019 [27]	RCT	Preterm babies between 23–37 weeks	Iran	Education and counselling intervention. Individual information giving and skills training, with materials, in care of infant. Delivered by a Researcher over 4 sessions (60–90 mins each) in NICU. Researcher available for additional telephone support, 2–4 days post-birth to discharge.	Usual care. Routine care did not include information on condition of infant and trained staff not always available to provide support.	Maternal anxiety at 1–2 months; assessed with State Trait Anxiety Inventory (STAI).	Y	Y	Y	UUU	Y	Y	Y	N	U	U	U
Newnham 2009 [28]	RCT	Preterm babies below 37 weeks	Australia	Education and counselling intervention. Individual information giving and skills training in infant care, development and sensitivity to infant. Delivered by a Researcher over 7 (30–60 min) sessions over final 2 weeks in NICU, 1 at home and 1 hospital visit, 2 weeks pre- to 3 months post-discharge.	Enhanced care. Received session 1 where discussions about their birth were had and they were assessed for depression, then the group had standard hospital care.	Infant temperament at 6 months; assessed by Short Temperament Scale. Mother-infant interaction at 6 months; assessed with: Synchrony Scale. Maternal depression at 6 months; assessed with EPDS.	Y	Y	Y	UUY	Y	Y	Y	Y	U	U	U
Pinelli 2001 [29]	RCT	Very low birth weight	USA	Education and counselling intervention. Individual skills training, with materials, in breastfeeding. Delivered by an Independent lactation consultant Weekly in NICU, ‘frequently’ at home after discharge, 72h following birth until 1 year or B/F discontinued.	Usual care. Standard breastfeeding support from regular staff members confined to the period of hospitalization in the NICU	Duration of exclusive breastfeeding.	Y	Y	Y	UUU	Y	Y	Y	N	U	U	U
Thakur 2012 [30]	RCT	Low birth weight	Bangladesh	Education and counselling intervention. Individual information giving in breastfeeding. Does not state who the intervention is delivered by but is delivered across 4 sessions (x2 per month from initiation of breastfeeding for 2 months).	Usual care. No details.	Infant weight and length at 60 days. Breastfeeding.	Y	Y	Y	UUU	Y	Y	Y	N	U	U	U
White Traut 2013 [31]	RCT	Preterm babies between 29–34 weeks	USA	Education and counselling intervention. Individual information giving and skills training, with materials, in infant development and sensitivity to infant. Delivered by a Research nurse, twice daily in NICU by mother or research nurse and continued at home by mother. Two facility sessions, two home visits and two telephone calls. Study entry (or 32 weeks) to 1-month post-discharge.	Enhanced care. Attention Control Condition’ a similar amount of contact with the mother and staff attention, but with no intervention content. Mothers received educational content that included premature infant care and car safety and four phone calls after the infant’s discharge to home. During each phone call, mothers received information on infant care including bathing, sleep positions and sleep habits, holding the baby, and safety of infant	Mother-infant interaction at 6 weeks; assessed with: Nursing Child Assessment Satellite Training–Feeding Scale (NCAST-Feeding).	Y	Y	Y	UUY	Y	Y	Y	Y	U	U	U
Wu 2014 [32]	RCT	Preterm & Very low birth weight	Taiwan	Education and counselling intervention. Individual information giving and skills training in infant care and development. Delivered by Nurse & physical therapist across 5 sessions NICU/ 8 home, within 7 days birth to 12 months CA.	Usual care consisting of child-focused in-hospital interventions and neonatal clinic visits. In-hospital interventions that addresses the integration between intra-organism subsystem functions and the sensory input from environment during the newborn period and emphasizes child-focused services including modulation of environment and teaching of child developmental skills.	Mother-infant interaction at 12 months follow-up; assessed with: Free-play procedure.	Y	Y	Y	UUY	Y	Y	Y	Y	U	U	U
Zelkowitz 2009 [33]	RCT	Very low birth weight	Canada	Education and counselling intervention. Individual information giving, with materials, in self-care and sensitivity to infant. Delivered by Nurse, psychologist/ graduate student trained to deliver intervention across 5 (1 hour sessions NICU) from average 33 days post-birth/ 1 home 2–4 weeks post- discharge.	Usual care. Control given general information about care	Maternal anxiety at 6 months; assessed with STAI.	Y	Y	Y	UUY	Y	Y	Y	Y	U	U	U
Mohammadian 2021 [34]	RCT	Preterm babies between 34–37 weeks	Iran	Education and counselling intervention. Individual information giving with breastfeeding, health conditions and skin to skin. Delivered by Nurse, Face to face plus telephone support. 14 days of daily continuous supportive counselling by telephone after neonatal discharge.	Usual care. No details.	Breastfeeding self-efficacy at 1, 2, 3, 4 months.	Y	Y	Y	NNN	Y	Y	Y	N	Y	Y	U
Korgali 2022 [35]	RCT	Preterm babies between 32–37 weeks	Turkey	Education and counselling intervention. Individual information giving. Delivered by a Paediatrician and paediatric nurse across 2-hour home visit one week after discharge then month 1, 2, 3 to give information about health of infant, breastfeeding and parent-baby relationship.	Usual care. Infants and families received routine follow-up in the Paediatric Polyclinic.	Breastfeeding, depression, anxiety, infant character perception scale at 1, 3, 6, 12 months corrected age.	Y	Y	Y	NNY	Y	Y	Y	N	Y	Y	U
Hwu 2023 [36]	RCT	Preterm babies between 32–37 weeks	Taiwan	Education and counselling intervention. Individual information giving, with detail and materials on massage and infant care delivered by Nurse and qualified lecturer across One session provided by qualified practitioner and asked to perform twice daily.	Usual care. Received guidance on premature infant care but did not perform infant massage.	Infant weight, parent attachment and stress at baseline at 1, 2, 4, 8 and 12 weeks.	Y	Y	Y	NNN	Y	Y	Y	Y	Y	Y	U
Hadi 2022 [37]	Quasi-exp	Low birth weight	Indonesia	Education and counselling intervention. Individual information giving, with materials on kangaroo care, breastfeeding and handwashing. Delivered by primary health centre nurses trained in LBW infant care across 3 sessions on the second and sixth week.	Enhanced care. Usual care plus information booklets	Breastfeeding, infant care practices, handwashing at 2, 6 and 12 weeks.	Y	N	Y	UUU	Y	Y	Y	N	Y	Y	U
Jang 2021 [38]	Quasi-exp	Preterm babies between 34–36 weeks	South Korea	Education and counselling intervention. Individual information giving, with materials on breastfeeding. Delivered by a Lactation consultant across 5 sessions once a week.	Usual care. The control group learned how to care for babies during visits.	Breastfeeding at 1, 2, 3 and 4 weeks.	Y	N	Y	UUU	Y	Y	Y	N	Y	Y	U
Akhbari Ziegler 2021 [39]	RCT	Preterm babies below 32 weeks	Switzerland	Education and counselling intervention. Individual information giving, with support from a coach at home to stimulate infant motor development. Delivered by a Coach, Weekly face to face sessions 30–45 mins duration at home for 6 months.	Usual care. One weekly face-to-face session lasting 30–45 minutes	Infant motor profile (median and range), family empowerment scale at 3, 6 months and 18 months corrected age.	Y	Y	Y	NNY	N	Y	N	N	Y	Y	U
Agrasada 2005 [40]	RCT	Low birth weight	Philippines	Home visits intervention. Individual information giving and skills training in breastfeeding. Delivered by Trained village volunteers across 8 home visits, 3 days following birth until 5.5 months.	Usual care (No home-visit programme)	Exclusive breastfeeding at 6 months.	Y	Y	Y	UUU	Y	Y	Y	N	Y	N	U
Baraldi 2024 [41]	RCT	Preterm babies born below 28 weeks	Sweden	Home visits intervention. Individual information giving and training using a strength-based intervention, positive parent–child interplay. 10 home visits.	Usual care. Extended treatment as usual including extra follow-up visits relating to this study	Neurodevelopmental (Emotional availability) at 12 months corrected age	Y	Y	Y	NUY	Y	Y	Y	Y	Y	U	U
Gardner 2003 [42]	RCT	Low birth weight	Jamacia	Home visits intervention. Individual information giving, materials in sensitivity to infant. Delivered by Community health workers across Weekly 1h home visits for 8 weeks.	Usual care. No details	Bayley scales of infant development; at 10–12 months.	Y	Y	N	UUU	Y	Y	Y	Y	U	U	U
Gunn 2000 [43]	RCT	Preterm babies below 37 weeks	Australia	Home visits intervention after early discharge. Individual breastfeeding support. Delivered by Home care nurse specialists with neonatal experience across Daily visits for 7–10 days post-discharge. Telephone support available	Usual care following discharge which included home Care Nurses made home visits or telephone contact during office hours on weekdays. Typically, this was daily for the first 5 weekdays after discharge, and then as necessary to support breastfeeding and other problems.	Number exclusively breastfeeding at 6 months	U	Y	Y	UUU	Y	Y	N	N	U	U	U
Eun Sun 2020 [44]	RCT	Preterm babies below 37 weeks	South Korea	Home visits intervention. Individual and group information giving and skills training in care of and sensitivity to infant. Delivered by an Experienced NICU nurse and a community visiting nurse across 1 or 2 home visits per month for 6 months, plus group support sessions.	Usual care of home visits by community nurses and no support groups or self-help meetings provided.	Maternal stress at 6 months; PSI.	Y	N	Y	UUU	Y	Y	Y	N	U	U	U
Koldewijn 2005, 2009 [45,46]	Before and after	Preterm babies below 32 weeks	Netherlands	Home visits intervention. Individual information giving, in care planning, infant development and sensitivity to infant. Delivered by Trained paediatric physical therapists across 6–8 (1h) home visits as required. 1-week post-discharge to 6 months.	Usual care. This included regular outpatient visits to the paediatrician. The local paediatricians were free to refer any infant for physical therapy if necessary; however, control infants were not allowed to be referred to an intervention-trained paediatric physical therapist	Infant temperament at 6 months; Infant behavioural assessment (IBA).	Y	Y	Y	UUY	Y	Y	Y	Y	U	U	U
Mazumder 2019 [47]	RCT	Low birth weight	India	Home visits intervention. Individual information giving and skills training in breastfeeding and KMC. Delivered by Intensively trained intervention worker (not HW) across 9 home visits (30–40mins) at 1–3, 5, 7, 10, 14, 21, 28 days.	Usual care. Consists of some home visits and breastfeeding techniques.	Infant mortality up to 180 days Number exclusively breastfeeding at 6 months.	Y	Y	Y	NNN	Y	Y	Y	Y	U	U	U
Meijessen 2010; 2011 [48,49]	RCT	Preterm or very low birth weight	USA	Home visits intervention. Individual information giving, in care planning, infant development and sensitivity to infant. Delivered by Experienced trained paediatric physical therapist across 6–8 (1h) home visits as required until 12 months CA.	Usual care. Consisted of regular visits to the outpatient local paediatric clinic	Maternal stress at 12 months; PSI.	Y	Y	Y	UYY	Y	Y	Y	N	U	U	U
Sinha 2021, 2022 [50,51]	RCT	Low birth weight	India	Home visits intervention. Individual information giving and skills training in breastfeeding and KMC. Delivered by Intensively trained intervention worker (not HW) across 9 home visits (30–40mins) at 1–3, 5, 7, 10, 14, 21, 28 days.	Usual care. Home-based postnatal care visit by accredited social health worker	Maternal depression at 28 days; Patient health questionnaire.	Y	Y	Y	NNN	Y	Y	Y	Y	U	U	U
Taneja 2020 [52]	RCT	Low birth weight	India	Home visits intervention. Individual information giving and skills training in breastfeeding and KMC. Delivered by Intensively trained intervention worker (not HW) across 9 home visits (30–40mins) at 1–3, 5, 7, 10, 14, 21, 28 days.	Usual care. Home Based Post Natal Care visits by government health workers as implemented through the health system	Cognitive development. Bayley scales of infant development; at 10–12 months.	Y	Y	Y	NNN	N	N	Y	Y	U	U	U
Youn 2021 [53]	RCT	Preterm babies below 30 weeks or VLBW	South Korea	Home visits intervention. Individual information giving and group skills training in infant care, development and sensitivity to infant. Delivered by Specialist nurse and physiotherapist (infant neurodevelopment) across 4 home visits (Discharge until 2 months CA) 12 x 90 min group sessions with physiotherapist.	Usual care. No home visits or group interventions.	Cognitive and motor development at 10 months;. Bayley scales of infant development Mother/infant attachment at 6 months; Mother-Child Attachment (MCA) scale.	Y	Y	Y	UUY	Y	Y	Y	N	U	U	U
Ingram 2016 [54]	Before and after	Preterm babies between 27–34 weeks	UK	Discharge preparedness intervention. Planning for early discharge. Care of infant. Delivered by NICU staff as required, for 5 weeks, between 27–33 gestation.	Usual care. No details.	Emergency hospital visits; number until 2 months.	Y	N	Y	NNN	Y	Y	Y	N	U	Y	U
Lee 2019 [55]	RCT	Preterm babies below 32 weeks	Hong Kong	Discharge preparedness intervention. Planning for discharge. Care of infant. Delivered by Neonatal nurse consultant across 3 sessions and 1 FU tele call. 34 gestation to 72h post-discharge.	Usual care. No details	Maternal stress 1–2 months; assessed with Perceived Stress Scale (C-PSS).	Y	Y	Y	UUY	Y	Y	Y	Y	U	U	U
Neyestani 2017 [56]	RCT	Preterm babies between 30–35 weeks	Iran	Discharge preparedness intervention. Planning for discharge. Care of infant. Materials. Included parent-directed contact following discharge if required. Delivered by Nurse across 4 (35–40 min) sessions plus 4 telephone contacts (x1 per week, 5–10 mins) after discharge. 48h from birth until 4wks post-discharge.	Usual care. No details	Maternal quality of life at 4 weeks; assessed with WHOQOL-BREF.	Y	Y	Y	UUU	Y	Y	N	N	U	U	U
Ortenstrand 2001 [57]	RCT	Preterm babies below 37 weeks	Sweden	Discharge preparedness intervention. Planning for discharge and early discharge. Care of infant. Delivered by Project nurse (neonatal trained) through Care planning session and domiciliary care visits (number unclear). Pre-discharge until domiciliary care completed.	Usual care. Discharge care supported by staff.	Maternal anxiety at 3 months; assessed with STAI.	Y	Y	Y	UUU	U	Y	Y	N	U	U	U
Ericson 2018 [58]	RCT	Preterm babies below 37 weeks	Sweden	Digital communication intervention. Individual support/communication in breastfeeding. Delivered by Breastfeeding support team (NICU staff trained for 2 days) through Daily telephone call from discharge for 14 days.	Usual care. Reactive telephone support.	Exclusive breastfeeding at 1–2 months.	Y	Y	Y	UYY	Y	Y	Y	Y	Y	U	U
Hagi-Pederson 2020 [59]	RCT	Preterm babies below 37 weeks	Denmark	Digital communication intervention. Individual support/communication in breastfeeding. Delivered by Nurse trained in early in-home care and trained in smartphone application, video consultations through 2–3 consultations per week by video following introduction in NICU, until discharge programme completion.	Usual care. In hospital consultations.	Exclusive breastfeeding at 1–2 months. Maternal-infant interaction at 1 month; assessed with: Mother and Baby Interaction Scale (MABISC).	Y	Y	Y	UUU	Y	Y	Y	N	U	U	U
Luu 2017 [60]	Before and after	Preterm babies below 30 weeks	Canada	Digital communication intervention. Individual support/communication, with materials, in infant care. Delivered by Certified occupational therapist trained in developmental care through 3x in-person workshop and 4 web-based modules (commenced in NICU), until 12 months.	Usual care. No web-based intervention.	Maternal-infant interaction at 4 months; assessed with: Parental Cognitions and Conduct Toward the Infant Scale.	Y	N	Y	NNN	Y	Y	Y	N	U	U	U
Robinson 2016 [61]	RCT	Preterm babies between 27–37 weeks	Sweden	Digital communication intervention. Individual support/communication in infant care. Delivered by developmental care team through 3x Skype calls per week with messaging option, to discharge from home health care.	Usual care. Families visit the neonatal nurse 2–3 times per week. Family could phone the nurse but did not receive the web-application.	Emergency hospital visits up to 2 months post-discharge.	Y	Y	Y	NNN	Y	Y	Y	N	U	U	U
Bahmanpour 2023 [62]	RCT	Preterm babies between 28–36 weeks	Iran	Digital communication intervention. Individual support and communication with materials in infant care. Delivered by Nurse through Daily education materials for 4 weeks. Social media following with educational files via mobile phone on care provision for newborn. Personal contact with researchers to review materials and answer questions.	Usual care. Routine treatment and follow-up.	Feelings of ‘hope’ as per the Hope Scale (mean and SD) and maternal perceived self-efficacy (PMPS-Q mean and SD) before the intervention, immediately after and at 4 weeks.	Y	Y	U	NNN	Y	Y	Y	N	Y	Y	U
Yan 2022 [63]	RCT	Preterm babies (no definition)	China	Digital intervention. Health education app on premature care and complications recognition. Online parent chat. Duration not reported.	Usual care. Routine outpatient follow-up management	Anxiety and depression at one month of age	Y	Y	Y	UYY	Y	Y	Y	Y	U	U	U
Zhang 2023 [64]	RCT	Preterm babies 28 – 36 + 6 weeks	China	Digital intervention. Online classes, counselling and chat platform	Usual care. Routine health education before discharge	Infant Developmental Scores	Y	Y	U	UUU	Y	Y	Y	Y	U	U	U
Neila-Vilen 2016 [65]	RCT	Preterm babies below 35 weeks	Finland	Peer support intervention. Individual support and materials in breastfeeding. Delivered by Volunteers with experience of B/F preterm infants (no training given). Midwife available to answer B/F questions, as required (facility/ home), to 12 months.	Usual care. Routine breastfeeding support provided.	Duration of exclusive breastfeeding.	Y	Y	Y	UUU	Y	Y	Y	Y	U	U	U

The quality of the studies included when critically appraised was unclear or low in many reported outcomes due to limited explanation of randomisation, allocation concealment and assessor blinding. Most studies were of low or very low certainty evidence based on GRADE, as a result of risk of bias, indirectness and imprecision (S1–S5 Tables), with the exception of and those utilising home-visit interventions reporting mortality, exclusive breastfeeding and cognitive development assessed at 10–12 months were graded as moderate certainty (S2 Table)

#### Types of interventions.

Five groups of interventions were identified: education and counselling interventions initiated in the health facility, home visits by a trained health worker or volunteer, discharge preparation and readiness interventions, digital communication interventions and peer support interventions. Although all interventions focused on providing support to participants to care for their infants in the home, there were similarities across some components of the interventions between groups (see S1 Fig), with many providing information, skills and support on an individual or group basis, or through different materials such as literature, workshops or lectures. Studies were grouped for analysis by the interventions that they shared the greatest number of intervention characteristics with (e.g., mostly home visiting or mostly digital communication); more detail is provided in Table 1. All the interventions commenced either in the health facility prior to discharge or in the home following birth or shortly after discharge from the facility. Interventions focused on mothers, parents and infants. Most control groups provided usual care, which varied according to settings, context, and by the nature of the support being offered. Six studies provided enhanced care in the control group [20, 22, 24, 28, 31, 37–] Table 1; denoted by *on forest plots). See S6 Table for a summary overview of the interventions.

*Education and counselling interventions initiated in the health facility:* Twenty-four studies included in this category focused on education or training of parents to care for their newborn; which was delivered on a one-to-one basis or with other parents in a group (14–39). The intervention training topics covered aspects of responsiveness and sensitivity to the infant, understanding and promotion of infant development, care of the newborn and breastfeeding. All provided education in the facility, with fifteen studies continuing education in the home following discharge, with interventions provided by health workers or researchers. Six studies in this group had enhanced care in the control groups [20, 22, 24, 28, 31, 37–].

*Home visiting interventions:* Eleven home visiting studies comprised of interventions which commenced and continued in the home following birth [40–5153]. Parents were supported in aspects of their infants care including care planning in the home, sensitivity and responsiveness, promotion of infant development and breastfeeding. The interventions were provided by healthcare workers, community health workers, trained intervention workers or trained volunteers.

*Strengthened discharge preparedness interventions:* Four studies focused on preparing parents for the discharge of their infant, in addition to normal discharge preparation [54–57]. This involved individualised discharge planning with the parents, along with advice on general care of the infant. The interventions commenced in the facility, with three continuing at home after discharge; two by telephone contact. These interventions were delivered by health workers trained in neonatal care.

*Digital communication interventions:* Seven studies comprised of interventions focusing on support and communication specifically through digital means, using web-based, Skype, apps or telephone media [58–64]. Parents were supported in care of the infant and breastfeeding, with interventions commencing either in the facility or at home. These were provided by health workers trained in delivery of the intervention, including neonatal intensive care unit (NICU staff), nurses and, in one study, occupational therapists, but were then self-directed.

*Peer support interventions:* One study comprised of interventions delivered by peer supporters who were women with experience of caring for a preterm infant in a similar environment and were willing to use their experiences to support others [65]. The focus was on general and breastfeeding support. This intervention commenced in the facility and took place following agreement from the mother or were initiated by the mother.

#### Outcomes.

Outcomes are reported in relation to each intervention type, with accompanying evidence tables.

### Education and counselling interventions initiated in the health facility

Twenty-four studies were included in this intervention category (14–39). Of these, fifteen reported infant outcomes including infant weight (three studies), infant length (three studies), exclusive breastfeeding (five studies) and cognitive development (four studies) (S1 Table).

**Fig 2:**
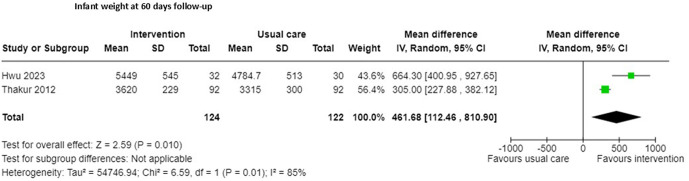
Infant weight at 60 days follow-up.

An improvement in infant growth measured by length and weight was reported in three RCTs [18,30,36], none of the studies reporting this outcome had enhanced care in the control group. An increase in length of the infant (measured in centimetres) in the intervention group at 60 days follow up (one study, n = 184, Mean Difference (MD) 1.5 cm, 95% Confidence Interval (CI) 1.1 cm to 1.9 cm; certainty of evidence very low [30]) and 120 days follow up (one study; n = 57, MD 1.2 cm, 95% CI 0.2 cm to 2.6 cm; certainty of evidence very low [18]) was evident, although detail was lacking on whether the participants and investigators were blinded to the allocation in both studies, but all study participants were accounted for [18,30]. Infant weight (measured in grams) also increased in the intervention groups at 60 days (MD 461 g, 95% CI 112 g to 811; certainty of evidence very low; Fig 2; S1 Table) in a meta-analysis of two studies [30,36] and a single study reported an increase in infant weight at 120 days (MD 410 g, 95% CI 406 g to 414 g; certainty of evidence very low) [18], similarly detail was lacking on blinding of the outcome assessors in one study [30], and did not take place in the remaining study [36]. One quasi-experimental study (n = 40) [38] found a small increase in infant length (MD 1.40 cm 95%CI 0.47 cm to 2.33 cm; certainty of evidence very low) and weight (MD 364g 95% CI 44g to 684g; certainty of evidence very low) at 60 days follow up.

**Fig 3:**
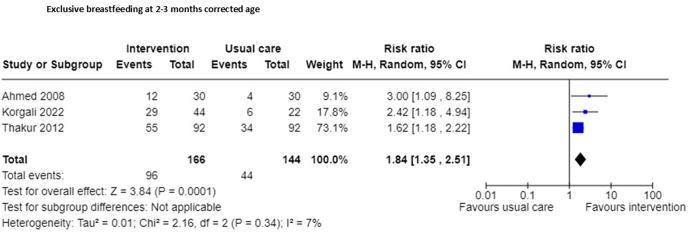
Exclusive breastfeeding at 2-3 months corrected age.

An increase in exclusive breastfeeding at 2–3 months corrected age was reported by three RCTs [14,30,35], favouring the intervention group (n = 310, RR 1.84, 95% CI 1.35 to 2.51; certainty of evidence very low; Fig 3; S1 Table), outcome assessors we blinded in one study [35], but this was not reported in two studies [14,30], however all participants were accounted for in all studies. One quasi-experimental study (n = 40) found that breastfeeding rates were higher in the intervention group at one month follow-up, when compared to the control group (RR 2.33 95% CI 1.26 to 4.30; certainty of evidence very low) [38]. None of the studies reporting this outcome had enhanced care in the control group.

**Fig 4:**
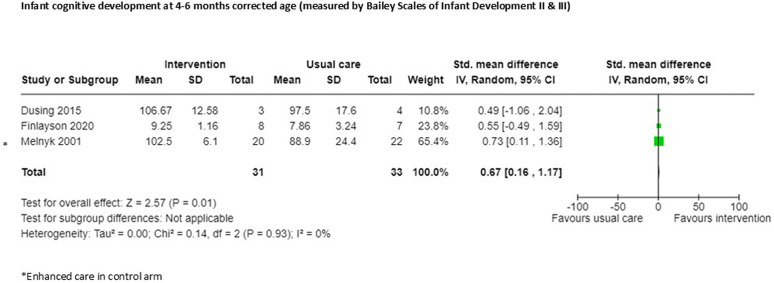
Infant cognitive development at 4-6 months corrected age.

Three small RCTs [24,17,19] reported cognitive development at 4–6 months corrected age and were included in meta-analysis, suggesting moderate evidence of effect for those infants receiving the intervention (n = 64, SMD 0.67, 95% CI 0.16 to 1.17: certainty of evidence very low; Fig 4; S1 Table). One of the studies in this meta-analysis had enhanced care in the control group [20]. All studies reporting this outcome in this analysis accounted for all participants, two studies blinded outcome assessors [24,17], but one study did not report this [19].

Maternal outcomes were reported by 11 studies and included maternal stress (three studies), depression (three studies), anxiety (six studies) and maternal-infant interaction (four studies) (S1 Table). Three RCTs [22,21,23] measured stress, at differing time points, with two studies reporting no evidence of effect at 15 days using the parental stress scale [22,23] (one study, n = 52, MD -1.12, 95% CI -14.32 to 12.08; certainty of evidence very low; this study provided enhanced care to the control group) or three months corrected age using the parenting stress index (one study, n = 199, MD 4.80, 95% CI -0.56 to 10.16; certainty of evidence low). However, one study [23] reported a reduction in stress at 12 months corrected age using the parental stress scale (n = 130, MD -13.70, 95% CI -25.5 to -1.89; certainty of evidence low).

**Fig 5:**
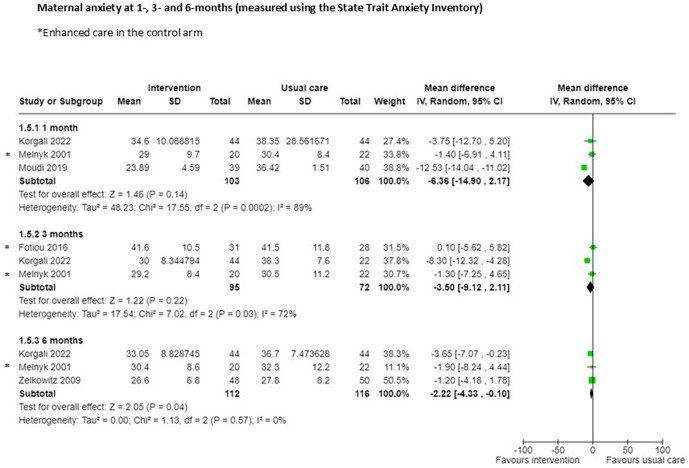
Maternal anxiety.

Six RCTs measured anxiety using the State Trait Anxiety Inventory (STAI) at different time points. A meta-analysis of three RCTs reporting no evidence of effect at one month (n = 209; MD -6.36 95%CI -14.90 to 2.17; certainty of evidence low; Fig 5) [24,27,35], or three months (n = 167; MD -3.50 95%CI -9.12 to 2.11; certainty of evidence very low; Fig 5; S1 Table) [24,35,66]. However, meta-analysis of three RCTs demonstrated a reduction in anxiety at six months follow-up with the intervention (n = 228; MD -2.22 95% CI -4.33 to -0.10; certainty of evidence low; Fig 5) [24,33,35]. A single study assessed this outcome at seven days. One of the study outcomes in these meta-analyses had enhanced care in the control group [20], three reported the blinding of outcome assessors [24,33,35], one did not blind assessors [20], and one did not report this [27].

**Fig 6:**
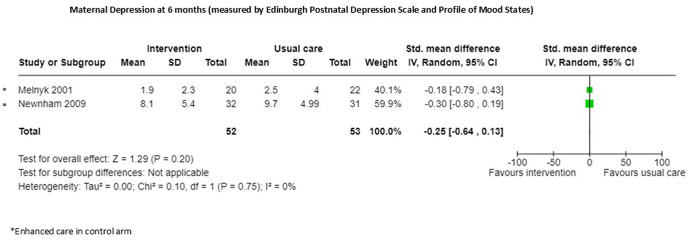
Maternal Depression at 6 months.

One RCT [22] reported no evidence of effect on maternal depression following a 15-day intervention (n = 52 MD -0.64, 95% CI -3.53 to 2.25). Meta-analysis of two studies [24,28] also demonstrated no effect on depression (n = 105 SMD -0.25, 95% CI -0.64 to 0.13; certainty of evidence low) at six months (Fig 6; S2 Table), however both studies in the group provided enhanced care in the control group, both blinded outcome assessors and accounted for all participants.

### Home visiting interventions

Eleven studies were included in this category [40–5153]. Nine studies reported infant outcomes; of these one reported mortality, three reported on exclusive breastfeeding, three studies reported cognitive outcomes, one reported a motor developmental outcome, and one study reported a temperament outcome (S2 Table).

One large RCT [47](n = 7479) reported a reduction in infant mortality in the intervention group (RR 0.71, 95% CI 0.57 to 0.89; certainty of evidence moderate) at 180 days follow up.

**Fig 7:**
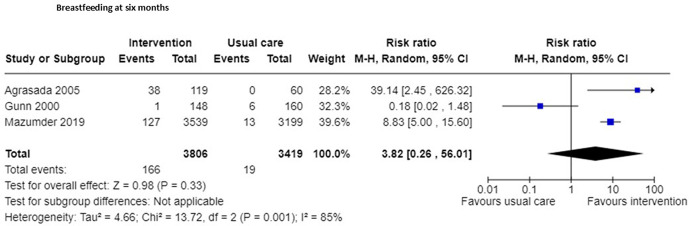
Exclusive breastfeeding at 6 months corrected age.

Three RCTs [40,43,47] included in a meta-analysis suggest no evidence of effect in exclusive breastfeeding at six months corrected age (n = 7225, RR 3.82, 95% CI 0.26 to 56.01; certainty of evidence moderate: Fig 7; S2 Table), two study outcomes did not report whether outcome assessors were blinded [40,43] and one study did not blind outcome assessors [47].

**Fig 8:**
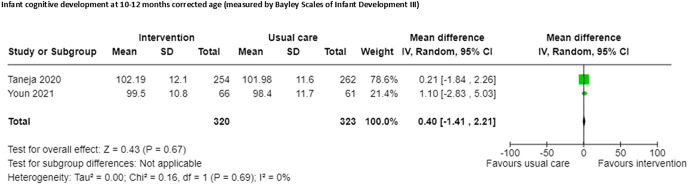
Infant cognitive development at 10-12 months corrected age.

Two RCTs [52,53] included in a meta-analysis reported no effect on cognitive development at 10–12 months corrected age and were included in meta-analysis (n = 643, SMD 0.40, 95% CI -1.41 to 2.21; certainty of evidence moderate: Fig 8; S2 Table), one study outcome blinded outcome assessors [53], but the other did not [52]. One study reported emotional availability at 12 months corrected age [41].

There was evidence of effect on motor development at 10 months corrected age in one RCT [53] (n = 136, MD 0.20, 95% CI 4.47 to 4.07; certainty of evidence low), but no evidence of effect for infant temperament [15] (n = 161, MD 0.70, 95% CI -0.6 to 1.46;) at six months corrected age.

Five RCTs reported maternal outcomes including maternal depression (two studies), stress (two studies) and mother-infant attachment (one study).

A reduction in maternal depression was reported at 28 days post-birth in one RCT [50] (n = 1147, RR 0.74, 95% CI 0.55 to 1.00; certainty of evidence low), assessors of this outcome were not blinded in this study. However, an RCT [53] reporting maternal depression at 6 months corrected age found no evidence of effect between groups (n = 136, MD-1.30, 95% CI -3.50 to 0.90; certainty of evidence low). Conversely there was no evidence of effect reported in one quasi-experimental study for maternal stress at 6 months [44] (n = 56, MD -3.54 95% CI -16.21 to 9.13; certainty of evidence very low) or by one RCT at 12 months [48] corrected age (n = 162, MD 1.69, 95% CI -3.18 to 6.56; certainty of evidence low) or mother-infant attachment [53] (n = 136, MD 1.20, 95% CI 2.79 to 0.39; certainty of evidence low) at 6 months corrected age.

### Strengthened discharge preparedness interventions

Four studies were included in this category [54–57], with the two maternal outcomes and one infant outcome reported.

One quasi-experimental study [54] reported that infants in the intervention group were less likely to attend for emergency care than those in the control group (n = 173, RR 0.62, 95% CI 0.39 to 1.0; certainty of evidence very low) at two months follow up (S3 Table), but this lacked randomisation and did not blind outcome assessors.

Three RCTs reported maternal outcomes including quality of life (one study) and stress (two studies) (S3 Table). All outcomes accounted for all participants but only one blinded outcome assessors [55]. One study [56] reported an increase in maternal quality of life in the intervention group (n = 56, MD 34.5, 95% CI 30.5 to 38.5; certainty of evidence very low) at four weeks follow up. Two RCTs reported no evidence of effect in maternal stress [55] (n = 26, MD -1.10, 95% CI -4.64 to 2.44; certainty of evidence low) at one to two months or anxiety [57] (n = 72, MD -2.30, 95% CI -5.49 to 0.89; certainty of evidence very low) at 3 months follow up.

### Digital communication interventions

A total of seven RCTs were included in this category [58–64], with three studies reporting infant outcomes of exclusive breastfeeding (two studies), emergency hospital visits (one study) and infant developmental scores at three months of age using the ages and stages questionnaire [64] (S4 Table). All but two studies accounted for all participants in this outcome [62], in which loss to follow-up was to reported, and two studies blinded outcome assessors [58,63]. Two studies reported no evidence of effect in exclusive breastfeeding [58,59] at one to two months post-discharge (n = 641, RR 0.96, 95% CI 0.84 to 1.10; certainty of evidence very low). One study reported emergency hospital visits [61] to be reduced in the intervention group (n = 89, median 0, range 0–7, compared with median 1 range 0–6) at 2 months post-discharge.

Two RCTs reported maternal-infant interaction as an outcome [59,60]. No evidence of effect was reported in either study at one month follow up [59] (n = 129, MD -0.80, 95% CI -1.84 to 0.24; certainty of evidence very low) or 4 months of age [60] (n = 85, MD -0.9, 95% CI -2.09 to 0.29; certainty of evidence very low), although there was significant loss to follow up.

### Peer support interventions

One study was included in this category [65], reporting one infant outcome of exclusive breastfeeding duration (one study) (S5 Table). This study did not report if outcome assessors were blinded, there was high loss to follow-up, but all participants were accounted for.

One RCT [65](n = 69) reported no evidence of effect in duration of exclusive breastfeeding between groups, with the intervention group reporting a median of 3 months (range 0–14 months) and the control group a median of 4.3 months (range 0–13 months) (certainty of evidence very low).

Formal exploration of the reasons for statistical heterogeneity by study features was limited owing to the small number of studies identified in our review. Given such clinical heterogeneity, it is unsurprising that statistical heterogeneity was identified in the analyses. No meta-analysis included 10 or more studies so funnel plots were not constructed.

### Outcome measurement scales

Several different measurement scales featured within this review to report the relevant outcomes.

#### Maternal outcomes.

Maternal anxiety, stress and depression were reported using ten different scales. Maternal anxiety was reported in six studies (four high-income countries and two middle-income country) and measured using two scales: the State Trait Anxiety Inventory (STAI); a validated scale for use in both settings [67,68] and the Self-Rating Anxiety Scale; also validated. Maternal stress was reported by six studies and measured using the perceived stress scale (two studies; one high income and one lower-middle income setting) and the perceived stress index (four studies; high income setting only); both scales are validated for use in these settings [69,70]. Maternal depression was reported in seven studies and measured using five different scales. The Patient Health Questionnaire was used in one study set in a lower-middle income setting, which is validated for use in many settings, including lower-middle income settings [71]. The Edinburgh Postnatal Depression Scale (EPDS) was used in one study, which was set in a lower-middle income setting (India), which is validated for use in such setting [72]. The Profile of Mood States (POMS) was used in two studies in high income settings and appropriately validated [73], one study using the WHOQOL-BREF (conducted in Iran) [74] and one study using the Centre for Epidemiologic Studies Depression Scale, a tool that has been validated in its setting south Korea [75]. The Self-Rating Depression Scale was used in a single study [63].

#### Perinatal outcomes.

Mother-infant attachment was reported by a single study and measured using the mother-infant attachment score [53]; but the scale used is not reported. Five studies report mother-infant interaction, and measure using five different scales. Two studies used the Nursing Child Assessment Satellite Training–Feeding Scale and the Nursing Child Assessment Teaching Scale, both of which have been validated for use in the given population [76]. One study set in Australia used the Synchrony Scale [28], a scale that has been used widely across multiple settings [77], and another in Taiwan assessed through modified Clark’s hierarchy of interaction behaviours [32].

One study set in Denmark used the Mother and Baby Interaction Scale (MABISC) [78]; and another set in Canada used the Parental Cognitions and Conduct Toward the Infant Scale (PACOTIS) [60]; both of which have been validated for use in similar settings [79,80]. Motor and cognitive development was reported in seven studies and was measured using the Bayley Scales of Infant Development I, II and III, the emotional availability scale and the ages and stages questionnaire. All studies except two [52,64] were set-in high-income settings [24,17,19,53], this scale has been validated for cross-cultural use [81]. Infant behavioural assessment (IBA) and temperament were assessed using two scales; two studies used the Short Temperament Scale in Australia [28,26], and one study used the Infant behavioural assessment [45] in the Netherlands.

## Discussion

This systematic review is the first, to our knowledge, to specifically address interventions to support parents in caring for preterm or LBW infants in the home. Whilst other reviews have focused on support during pregnancy for women and risk of low birth weight babies [82] or developmental interventions for preterm infants [83], this review specifically focuses on interventions to support families and care givers of preterm or low-birthweight babies in the home, appreciating this is a complex and stressful time.

The early period following babies’ transfer into the community is a critical period for all babies; preterm and low birthweight babies are particularly vulnerable [84], thus parental support for caring for these babies is important [85]. This review identified multiple interventions aimed at offering such support through information, skills or in-person or remote support (see S1 Fig), with evidence of effect related to some maternal and infant outcomes (see S2 Fig). Studies reporting education and counselling interventions demonstrated improvements in infant growth, exclusive breastfeeding, infant cognitive development, and reduced maternal stress, however the effect size may have been reduced due to the strengthening that took place in some of the control groups through enhanced care provision, or information detail lost through the group approaches often used. Discharge preparedness interventions reported evidence of effect for improved maternal quality of life. Home visiting interventions, often providing individualised information and support, demonstrated evidence of effect in reduction in infant mortality, increase in immunisation uptake, and reduction in maternal depression, whilst peer-support interventions noted evidence of effect in reduction in maternal anxiety. These findings support the need for greater support for women and families, and the broader caregiving unit in caring for their babies [86], as research has shown that involving community support mechanisms can have significant maternal and infant impact [87]. However, the wide variation in interventions, the methodological weaknesses and inconsistencies in outcome measures and scales highlights the need for further rigorous research prior to implementation of specific interventions.

Furthermore, although the findings are encouraging, none of the papers provided detailed process evaluation, therefore it is difficult to understand the mechanisms of effect of the interventions or the impact of contextual factors [88], making replicability challenging. Intervention fidelity and compliance were also generally ill-defined, with potential impact on outcomes [88]. Descriptions of usual care provided in control groups was often limited and likely to vary across different settings. Inconsistencies in outcome measures used and the timing of assessments prevented meta-analysis of data for the majority of papers. This suggests that, for this area of investigation, development of core outcome sets and consensus of key outcome measurement points would be valuable, to enable consensus on a standardised minimal group of important outcomes [89].

Parental acceptability of the interventions received little attention in the included studies with only six papers reporting any aspects of acceptability [43,54,60,61,65]. Where this was reported, the overall feedback from parents was positive. However, understanding acceptability is complex and dependent on several factors [90], and future studies should consider mixed methods of data collection, including in-depth qualitative explorations to capture the nuances of acceptability. For example, peer support was particularly well received [65], but it is difficult to determine whether this was a result of the intervention per se or because of the additional social contact with other mothers. Moreover, none of the papers reported involving women or families in the development of interventions. Engagement and involvement of women, families and communities provides for more relevant and sustainable interventions [91], and may result in greater impact [87].

The evidence presented in the included papers failed to provide confidence in the impact of the interventions on maternal psychological morbidity. Nevertheless, we know that outcomes such as stress, anxiety and depression are important to women when caring for their babies [7] suggesting further adequately powered, rigorous studies are required.

Although 47 studies were included in this review this was over a 24-year period with a diversity of interventions across high, upper-middle and lower-middle income settings. Whilst a plethora of research has been conducted to prevent preterm or LBW infants [92], or to provide support and care in the hospital [93], support for parents to care for their preterm or LBW infants at home has been a neglected area. In many settings there is limited or no postnatal support for women after discharge regardless of infant gestation or weight [94]. This is a particularly difficult time for parents, who have reported feeling vulnerable and inadequate in caring for their newborn when transitioning from a facility where support was available to being left to cope alone [6]. It is conceivable that a well-developed programme of support, in the home, to enable families to care for preterm or LBW infants may improve health outcomes for the infant and family, providing parents with the confidence and skills to care for their infant. The evidence to support such a programme needs further investigation, especially in low-income settings where evidence is lacking.

### Strengths and limitations

This is the first review exploring multiple interventions for parental support of preterm and LBW babies in the home, despite most of the outcomes focusing on maternal or infant effect. The review highlights the gaps in the evidence and illuminates the urgent need for further research in this area, in low-income settings where most of the vulnerable newborns reside. This review encompassed a detailed search across multiple databases with no restrictions, reducing the likelihood of studies being missed. After registration and discussion with experts, the review evolved to include interventions which were initiated in the facility or in the home, thus ensuring that the true nature of existing interventions was represented.

### Limitations of the evidence included in the review

Few studies provided data from low-middle income settings, and no studies provided data from low-income settings. The interventions themselves were disparate, with differences in intervention components (which, in some cases, were poorly described), methods of delivery and length and frequency of intervention. Moreover, outcome measures and timepoints at which these were measured also varied considerably between studies (see S3 Fig), which may inherently bias the outcome (e.g., postnatal depression scores may be affected by sleep regression). In addition to this, this study was limited by the exclusion of qualitative data which may have provided crucial insights into contextual, cultural, and practical aspects of providing care at home, as well as caregiver perspectives on acceptability and feasibility of interventions. Further reviews would benefit from the inclusion of qualitative studies.

### Limitations of the review process

This review was further impacted by the limited opportunities to synthesise the data. Due to the small number of studies within each analysis, we were not able to formally explore publication bias, or the statistical heterogeneity seen within our analyses.

Moreover, this review was limited by the methodological weakness of many of the included studies. Most included outcomes had small sample sizes, with limited or no discussion of sample size calculation. Risk of bias was high, with unclear or inadequately described randomisation and allocation concealment in some studies. Participant blinding was not possible in any of the included studies and assessor blinding was unclear in some outcomes. The overall certainty of evidence based on Grade was rated as very low or low, with only three outcomes including evidence of moderate quality (exclusive breastfeeding, infant mortality, cognitive development), all related to home visiting interventions. Outcomes were mainly downgraded for risk of bias, indirectness and imprecision as a result of methodological issues, single site/setting and sample.

## Conclusion

This review highlights the need for well-designed, effective support interventions, prioritised and developed with women, families and stakeholders. This will enable suitably tailored interventions which meet the needs of women and families, whilst improving infant outcomes. Components need to be carefully considered and evaluated in context, along with economic evaluation, and assessed for suitability for implementation in low-resource settings. Importantly, acceptability of interventions for women and families is vital in ensuring their success. Process evaluation and economic evaluation will also add valuable understanding to development and potential implementation of interventions in diverse settings, whilst qualitative research is needed to explore why interventions do not improve maternal wellbeing. Furthermore, given the disparity of outcomes measured, development of a core outcome set for support interventions should be considered, along with participatory approaches define what types of support women and parents actually value and need at home at this complex time. The evidence does confirm the viewpoint that support interventions for parents to care for preterm or LBW infants in the home may improve outcomes. This is an area of research which requires further exploration, as outlined above, in order to further improve outcomes for preterm of LBW infants, especially in low income setting where health systems may be fragile and poorly resourced.

**Strengths and limitations of this review**

- This is the first review exploring multiple interventions for parental support of preterm and LBW babies in the home.
- Meta-analysis was limited due to the heterogeneity of study interventions and outcome measures.
- The majority of studies were graded as low or very low certainty evidence as a result of risk of bias, indirectness and imprecision.

## Supporting information

S1 Text Search terms. (DOCX)

S1 Table Summary of findings for education and counselling interventions. (DOCX)

S2 Table Summary of findings for home visiting interventions. (DOCX)

S3 Table Summary of findings for strengthened discharge preparedness interventions. (DOCX)

S4 Table Summary of findings for digital communication interventions. (DOCX)

S5 Table Summary of findings for peer support interventions. (DOCX)

S6 Table Table of interventions. (DOCX)

S1 Fig Conceptual model of interventions. (TIF)

S2 Fig Conceptual model of outcomes. (TIF)

S3 Fig Visual mapping intervention types to outcomes assessed. (TIF)
