# Supplementary material for: Interventions to support parents, families and caregivers in caring for preterm or low birth weight infants at home: A systematic review and meta-analysis
Source: PLOS Glob Public Health. 2026 Feb 10;6(2):e0005690. doi: 10.1371/journal.pgph.0005690 (PMC12890145; doi:10.1371/journal.pgph.0005690)
Supplement: S1 Table — (DOCX) [file pgph.0005690.s002.docx]

**S1 Table: Education and counselling interventions**

| Outcomes | **Mean (SD) or No. (%)** | | **Relative effect (95% CI)** | **№ of participants (studies)** | **Certainty of the evidence (GRADE)** |
| --- | --- | --- | --- | --- | --- |
|  | **usual care** | Facility-initiated education and counselling |  |  |  |
| **Infant length**  **at 30 days**  follow-up (cm); Mean (SD) | 50.0 (1.5) | 51.4 (1.5) | MD 1.40 cm higher (0.47 cm higher to 2.33 cm higher) | 40  (1 non-randomised-exp) | ⨁◯◯◯ Very low ^b, c, d, e, i^ |
| **Infant length  at 60 days**  follow-up (cm); Mean (SD) | 48.7 (1.6) | 50.2 (1.3) | MD 1.5 cm higher (1.1 cm higher to 1.9 cm higher) | 184  (1 RCT) | ⨁◯◯◯ Very low ^a, b, c, e^ |
| **Infant length  at 120 days**  follow-up (cm); Mean (SD) | 58.6 (2.6) | 59.8 (2.8) | MD 1.2 cm higher  (0.2 cm higher to 2.6 cm higher) | 57  (1 RCT) | ⨁◯◯◯ Very low ^a, c, e,^ |
| **Infant weight**  **at 30 days**  follow up (g); Mean (SD) | 3,483 (576) | 3,847 (441) | MD 364g higher (44g higher to 684 higher) | 40  (1 non-randomised-exp) | ⨁◯◯◯ Very low ^b, c, d, e, h, i^ |
| **Infant weight  at 60 days**  follow-up (g); Mean (SD) | NA | NA | MD 461 g higher (112 g higher to 811 g higher) | 246  (2 RCT) | ⨁◯◯◯ Very low ^a,b,c,e,h^ |
| **Infant weight at 120 days**  follow-up (kg); Mean (SD) | 5.24 (0.95) | 5.56 (0.92) | MD 410 g higher (406 g higher to 414 g higher) | 57  (1 RCT) | ⨁◯◯◯ Very low ^a,c, e^ |
| **Exclusive breastfeeding** at 2-3 months; n (%) | 44/144 (30.6%) | 96/166 (57.8%) | RR 1.84 (1.35 to 2.51) | 310 (3 RCTs) | ⨁◯◯◯ Very low ^a,b,c,h^ |
| **Duration of exclusive breastfeeding**; Mean (SD) | 24.2 weeks (21.6 weeks) | 26.2 weeks (21.6 weeks) | MD 2.0 weeks higher (5.48 weeks lower to 9.48 weeks higher) | 128  (1 RCT) | ⨁◯◯◯ Very low ^b, d, e^ |
| **Breastfeeding at 1 month**  follow-up | 7/19 | 18/21 | R 2.33 (1.26 to 4.30) | 40  (1 non-randomised-exp) | ⨁◯◯◯ Very low ^b, c, d, e, i^ |
| **Motor development at 6 months**; assessed with Bayley Scales of Infant Development (BSID); Mean (SD) | NA | NA | SMD 0.38 higher (1.15 lower to 1.91 higher) | 7 (1 RCT) | ⨁◯◯◯ Very low ^a,d,e,f^ |
| **Cognitive development at 4-6 months**; assessed with BSID (II or III) Mean (SD) | NA | NA | SMD 0.67 higher (0.16 higher to 1.17 higher) | 64 (3 RCTs) | ⨁◯◯◯ Very low ^a, d, e^ |
| **Infant temperament at 6 months**; assessed by Short Temperament Scale   - Approach - Rhythmicity - Cooperation/manageability - Activity/reactivity - Irritability - Easy–difficult scale - Compare question - Approach - Infant colic - Infant sleep difficulties - Infant crying | 3.5 (0.50)  3.8 (0.57)  2.2 (0.63) 3.7(0.47)  3.4 (0.48)  3.0 (0.34)  2.30 (2.03–2.76) 2.42 (2.03–2.76) 0.95 (0.67)  0.58 (0.44)  0.89 (0.91) | 3.4 (0.39)  3.7 (0.55)  2.3 (0.51)  3.8 (0.53)  3.4 (0.36)  3.0 (0.31)  2.84 (2.52–3.03)  2.54 (1.86–2.37)  0.12 (0.10)  0.25 (0. 63)  0.55 (0.63) | MD -0.10 [-0.28 , 0.08]  MD -0.10 [-0.33 , 0.13]  MD 0.10 [-0.13 , 0.33]  MD 0.10 [-0.10 , 0.30]  MD 0.00 [-0.17 , 0.17]  MD 0.00 [-0.13 , 0.13]  MD 0.54 [0.11 , 0.97]  MD 0.12 [-0.31 , 0.55]  MD -0.83 [-1.06 , -0.60]  MD -0.33 [-0.59 , -0.07]  MD -0.34 [-0.71 , 0.03] | 92  (1 RCT)  63  (1 RCT) | ⨁◯◯◯ Very low ^a, d, e^ |
| **Mother-infant interaction at 6** **weeks**; assessed with: Nursing Child Assessment Satellite Training–Feeding Scale (NCAST-Feeding); Mean (SD) | 62.5 (7.0) | 64.3 (5.2) | MD 1.80 higher (0.21 higher to 3.81 higher) | 142  (1 RCT) | ⨁◯◯◯ Very Low ^d,e^ |
| **Mother-infant interaction** **at 3** **months**; assessed with: Nursing Child Assessment Teaching Scale (NCATS); Mean (SD) | 37.4 (4.9) | 36.6 (5.1) | MD 0.80 (2.20 higher to 0.60 higher) | 196  (1 RCT) | ⨁◯◯◯ Very Low ^d,e^ |
| **Mother-infant interaction at 6 months**; assessed with: Synchrony Scale; Mean (SD) | 0.24, (0.13) | 0.45 (0.08) | MD 21.0 (0.11 higher to 0.67 higher) | 63  (1 RCT) | ⨁◯◯◯ Very Low ^d,e^ |
| **Mother-infant interaction at 12** months follow-up; assessed with: Free-play procedure; Mean (SD)   - High quality maternal behaviour        - Engaged infant behaviour      - Synchronous didactic behaviour | 0.41 (0.27))  0.85 (0.19)  0.35 (0.25) | CHIB: 0.51 (0.28) HBIP: 0.46 (0.29)  CBIP 0.86 (0.13) HBIP 0.89 (0.15)  CBIP 0.44 (0.24) HBIP 0.42 (0.28) | MD 0.10 higher (0.01 lower to 0.21 higher)  MD 0.01 lower (0.06 lower to 0.08 higher)  MD 0.09 higher (0.01 lower to 0.19 higher). | (93)  1 RCT | ⨁⨁◯◯ Low ^c,e^ |
| **Maternal stress at 15 days** follow-up; assessed with parental stress scale (PSS) | NA | NA | MD 1.12 lower (14.32 lower to 12.08 higher) | 52  (1 RCT) | ⨁◯◯◯ Very Low ^b,d^ |
| **Maternal stress at 3 months** follow-up; assessed with Parenting Stress Index (PSI) | NA | NA | MD 4.80 higher (0.56 lower to 10.16 higher) | 199  (1 RCT) | ⨁⨁◯◯ Low ^b,d^ |
| **Maternal stress at 12 months** follow-up; assessed with PSI | NA | NA | MD 13.70 lower (25.5 lower to 1.89 lower) | 130  (1 RCT) | ⨁⨁◯◯ Low ^d,e^ |
| **Maternal anxiety at 7 days;**  assessed with State Trait Anxiety Inventory (STAI)  BEIGY 2021 | 53.85 ± 4.02 | 52.94 ± 8.05 | MD 0.91 lower (4.44 lower to 2.62 higher) | 50 (1 RCT) | ⨁◯◯◯ Very low ^b, c,d,h^ |
| **Maternal anxiety at 1 month**; assessed with State Trait Anxiety Inventory (STAI) | NA | NA | MD 6.36 lower (14.90 lower to 2.17 higher) | 209  (3 RCTs) | ⨁⨁◯◯ Low ^c,g,h^ |
| **Maternal anxiety at 3 months**; assessed with State Trait Anxiety Inventory (STAI) | NA | NA | MD 3.50 lower  (9.12 lower to 2.11 higher) | 167  (3 RCT) | ⨁◯◯◯ Very low ^b, c,d,h^ |
| **Maternal anxiety at 6 months**; assessed with STAI | NA | NA | MD 2.22 lower (4.33 lower to 0.10 lower) | 206  (3 RCT) | ⨁⨁◯◯ Low ^c,g,h^ |
| **Maternal depression at 15 days**; assessed with Edinburgh Postnatal Depression Scale (EPDS) | NA | NA | MD 0.64 lower (3.53 lower to 2.25 higher) | 52  (1 RCT) | ⨁◯◯◯ Very Low ^a,b,c,e^ |
| **Maternal depression at 6 months**; assessed with Profile of Mood States (POMS)/EPDS) | NA | NA | SMD 0.25 lower (0.64 lower to 0.13 higher) | 105  (2 RCTs) | ⨁◯◯◯ Very Low ^a, b,d^ |

a. Risk of bias, randomisation and/or allocation concealment unclear or not described. b. Risk of bias, blinding of assessors unclear c. Indirectness, low, lower-middle and upper or high income setting only. d. Imprecision, small sample. e. Indirectness, single study. f. Indirectness, attrition >10%. g. Risk of bias, randomisation and allocation concealment unclear in one study. h. Imprecision, heterogeneity, high I2 or wide variation in CI between studies. i. No randomisation
