# Supplementary material for: Interventions to support parents, families and caregivers in caring for preterm or low birth weight infants at home: A systematic review and meta-analysis
Source: PLOS Glob Public Health. 2026 Feb 10;6(2):e0005690. doi: 10.1371/journal.pgph.0005690 (PMC12890145; doi:10.1371/journal.pgph.0005690)
Supplement: S2 Table — (DOCX) [file pgph.0005690.s003.docx]

**S2 Table: Home visiting interventions**

| Outcomes | **Absolute effects** | | Relative effect (95% CI) | № of participants (studies) | Certainty of the evidence (GRADE) |
| --- | --- | --- | --- | --- | --- |
|  | **Usual care** | **Home visits** |  |  |  |
| Mortality until 180 days | 47 per 1,000 | 35 per 1,000 (28 to 44) | RR 0.71 (0.57 to 0.89) | 7479 (1 RCT) | ⨁⨁⨁◯ Moderate^g,e^ |
| Exclusive breastfeeding at 6 months | 19/3419 (0.6%) | 161/3806 (4.4%) | RR 3.82 (0.26 to 56.1) | 7221 (3 RCTs) | ⨁⨁⨁◯ Moderate^a,b^ |
| Immunization visits in the first year of life; Mean (SD) | 2.53 (0.11) | 3.74 (0.09) | MD 1.21 higher  (0.93 higher to 1.49 higher) | 970  (1 observational study) | ⨁◯◯◯ Very low^,c,d,e^ |
| Hospitalization until 12 months FU; mean (SD) | 0.25 (0.88) | 0.59 (1.76) | MD 0.34 higher  (0.16 higher to 0.52 higher) | 970  (1 observational study) | ⨁◯◯◯ Very low^c,d,e^ |
| Cognitive development at 10-12 months; assessed by BSID | NA | NA | MD 0.40 higher (-1.41 lower to 2.21higher) | 643  (2 RCTs) | ⨁⨁⨁◯  Moderate^a,b^ |
| Emotional availability at 12 months  Sensitivity  Structuring  Non-intrusiveness  Non-hostility  Child responsiveness  Child involvement | 25.9 (3.3)  25.8 (3.5)  25.5 (2.9)  27.1 (2.2)  25.5 (3.1)  24.3 (3.5) | 26.1 (2.4)  26.3 (2.0)  25.4 (2.9)  27.0 (1.9)  25.7 (1.9)  24.9 (2.4) | MD 0.20 (-0.85 to 1.25)  MD 0.50 (-0.04, 1.04)  MD -0.10 (-1.16 , 0.96)  MD -0.10 (-0.85 , 0.65)  MD 0.20 (-0.73 , 1.13)  MD 0.60 (-0.49 , 1.69) | 130 (1 RCT) | ⨁⨁◯◯ Low^e,f^ |
| Motor development  at 10 months; assessed by BSID | NA | NA | MD 0.20 lower (4.47 lower to 4.07 higher) | 136 (1 RCT) | ⨁⨁◯◯ Low^e,f^ |
| Infant temperament at 6 months; assessed by Infant behavioural assessment (IBA) | NA | NA | MD 0.70 higher  (0.6 lower to 1.46 higher) | 161  (1 RCT) | ⨁⨁◯◯ Low^f,e^ |
| Mother-infant attachment (MCA); at 6 months Mean (SD) | 101.3 (5.1) | 100.1 (4.3) | MD 1.20 higher  (2.79 higher to 0.39 higher) | 136  (1 RCT) | ⨁⨁◯◯ Low^e,f^ |
| Maternal stress at 6 months; assessed with PSI. Mean (SD) | 80.81 (24.11) | 77.27 (24.24) | MD 3.54 lower  (16.21 lower to 9.13 higher) | 56  (1 observational study) | ⨁◯◯◯ Very low^e,f,h^ |
| Maternal stress at 12 months; assessed with PSI | N/A | N/A | MD 1.69 higher (3.18 lower to 6.56 higher) | 162  (1 RCT) | ⨁⨁◯◯ Low^e,f^ |
| Maternal depression at 28 days (PT and LBW); assessed by Patient Health Questionnaire – 9 | N/A | N/A | RR 0.74 (0.55 to 1.00) | 1147  (1 RCT) | ⨁⨁◯◯ Low^e,g^ |
| Maternal depression at 6 months; assessed | N/A | N/A | MD 1.30 lower (3.50 lower to 0.90 higher) | 136  (1 RCT) | ⨁⨁◯◯ Low^e,f^ |

a. Risk of bias, randomisation and allocation concealment not described, in one study. b. Risk of bias, blinding of assessors not clear, in one study. c. Non-randomised, quasi-experimental study. Matched control group. d. Indirectness, single high-income setting, six sites. e. Single study. f. Indirectness, high-income setting. g. Indirectness, lower middle-income setting. h. Non-randomised, non-equivalent control group
