# Supplementary material for: Interventions to support parents, families and caregivers in caring for preterm or low birth weight infants at home: A systematic review and meta-analysis
Source: PLOS Glob Public Health. 2026 Feb 10;6(2):e0005690. doi: 10.1371/journal.pgph.0005690 (PMC12890145; doi:10.1371/journal.pgph.0005690)
Supplement: S4 Table — (DOCX) [file pgph.0005690.s005.docx]

**S4 Table: Digital communication interventions**

| Outcomes | **Effects** | | Relative effect (95% CI) | № of participants (studies) | Certainty of the evidence (GRADE) |
| --- | --- | --- | --- | --- | --- |
|  | **usual care** | **Digital communication** |  |  |  |
| **Exclusive breastfeeding at 1-2 months** | 207/361 | 185/327 | RR 0.96 (0.84 to 1.10) | 641 (2 RCTs) | ⨁◯◯◯ Very low^a,b,c^ |
| **Emergency hospital visits up to 2 months** post-discharge. Median (range) | Median 1  (0-6) | Median 0  (0-7) |  | 89  (1 RCT) | ⨁◯◯◯ Very low^a,c,d^ |
| **Maternal-infant interaction at 1 month** follow-up; assessed with: Mother and Baby Interaction Scale (MABISC); Total score, Mean (SD**)** | 11.3 (3.4) | 10.5 (3.1) | MD 0.80 lower  (1.84 lower to 0.24 higher) | 129  (1 RCT) | ⨁◯◯◯ Very low^a,c,e^ |
| **Maternal-infant interaction at 4 month** follow-up; assessed with: Parental Cognitions and Conduct Toward the Infant Scale (PACOTIS); Median (IQR) | 9.0 (7.2-10.0) | 8.1 (7.0-9.8) | MD -0.9 lower (-2.09 lower to –0.29 higher)  P 0.59). | 85  (1 non-randomised exp) | ⨁◯◯◯ Very low^c, f^ |
| **Development**  **Zhang 2023** | 191.00 (46.41) | 245.50 (21.27) | MD 54.50 higher (33.17 higher to 75.83 higher) | 44  (1 RCT) | ⨁◯◯◯ Very low^a,c,d^ |
| **Anxiety at 1 month**  Assessed with the self-assessed anxiety scale  **Yan 2022** | 60.2 (10.8) | 49.6 (8.5) | MD -10.60 lower (-13.71 lower to -7.49 lower) | 150  (1 RCT) | ⨁⨁◯◯ low^,b,c,^ |
| **Depression at 1 month**  Assessed with the self-assessed depression scale  **Yan 2022** | 58.8 (11.2) | 48.2 (9.5) | MD -10.60 lower (-13.92 lower to-7.28 lower) | 150  (1 RCT) | ⨁⨁◯◯ lowb^,b,c,^ |

a. Risk of bias, blinding of assessors not clear in one study b. Imprecision, small sample size in one study c. Indirectness, single high-income or low-middle setting d. Risk of bias, randomisation and allocation concealment unclear/not described e. Imprecision, high attrition >10% f. non-randomised, historical comparison group
