# Supplementary material for: Interventions to support parents, families and caregivers in caring for preterm or low birth weight infants at home: A systematic review and meta-analysis
Source: PLOS Glob Public Health. 2026 Feb 10;6(2):e0005690. doi: 10.1371/journal.pgph.0005690 (PMC12890145; doi:10.1371/journal.pgph.0005690)
Supplement: S5 Table — (DOCX) [file pgph.0005690.s006.docx]

**S5 Table: Peer support interventions**

| Outcomes | **Effects** | | Relative effect (95% CI) | № of participants (studies) | Certainty of the evidence (GRADE) |
| --- | --- | --- | --- | --- | --- |
|  | **usual care** | **Facility-initiated peer support** |  |  |  |
| Duration of exclusive breastfeeding; Median (range) | 4.3 months (range 0-13) in the control group. | 3 months (range 0-14) in the intervention group | - | 69  (1 RCT) | ⨁◯◯◯ Very low^a,b^ |

a. Risk of bias, unclear if outcome assessors blinded. b. Single high-income setting c. non-randomised cohort study with control group, >10% attrition
