# Supplementary material for: Interventions to support parents, families and caregivers in caring for preterm or low birth weight infants at home: A systematic review and meta-analysis
Source: PLOS Glob Public Health. 2026 Feb 10;6(2):e0005690. doi: 10.1371/journal.pgph.0005690 (PMC12890145; doi:10.1371/journal.pgph.0005690)
Supplement: S6 Table — (DOCX) [file pgph.0005690.s007.docx]

**S6 Table: Summary of interventions**

| Author | Population | Intervention type | Intervention components and focus | Length of intervention | Delivered by | Outcome & measure |
| --- | --- | --- | --- | --- | --- | --- |
| Ahmed 2008  (20) | P <37 | Education and counselling | Individual information giving and skills training in breastfeeding. | 4 in NICU /1 home, birth to 3 months post-discharge. | Researcher. | Exclusive breastfeeding at 2-3 months. |
| Beigy 2021  (21) | P 28 – 36 | Education and support intervention. | Knowledge and skills based on the mothers’ needs, awareness, and skill in caring for their infant during two home visits. | Two visits for seven days | Researcher | Anxiety (State – trait scale) 7 days after discharge |
| Dusing 2015, 2018  (22, 23) | P <37 | Education and counselling | Individual skills training in infant care and development. | At least twice in NICU/ twice weekly at home from discharge, birth to 3 months CA. | Trained and experienced NICU Physical therapist. | Motor and cognitive development at 6 months; assessed with Bayley Scales of Infant Development (BSID). |
| Fan 2021  (24) | P 28-32 | Education and counselling | Individual information giving and skills training in infant development. | 1 training lecture and 3 workshops in the facility with parents performing EI in the home, for 30 days. | Researcher with rehabilitation background. | Infant weight and length at 120 days follow-up. |
| Finlayson 2020  (25) | P <30 | Education and counselling | Individual skills training in infant care and development. | 5 in NICU/ 5 home, birth to 3 months CA. | Experienced Physiotherapist/ occupational therapists trained in intervention. | Cognitive development at 4-6 months; assessed with BSID. |
| Fotiou 2016  (16) | P <37 | Education and counselling | Group information and skills training, with additional materials, in self-care. | 5 sessions (90 minutes each) in NICU, parents practised independently for 3 months after discharge, birth to 3 months CA. | Researcher. | Maternal anxiety at 1-2 months; assessed with State Trait Anxiety Inventory (STAI). |
| Glazebrook 2007  (26) | P <32 | Education and counselling | Individual information giving and skills training in sensitivity to infant. | Weekly one-hour sessions until discharge / optional to continue until 6 weeks following discharge. | NICU nurse trained in intervention. | Mother-infant interaction at 3 months; assessed with: Nursing Child Assessment Teaching Scale (NCATS). Maternal stress at 3 months follow-up; assessed with Parenting Stress Index (PSI). |
| Jaywant 2020  (17) | P 28-36 | Education and counselling | Individual information giving and skills training in infant care and sensitivity to infant. | Daily session in NICU, for 15 days. | Therapist. | Maternal stress at 15 days follow-up; assessed with parental stress scale (PSS). Maternal depression at 15 days; assessed with Edinburgh Postnatal Depression Scale (EPDS). |
| Kaaresen 2005  (27) | LBW | Education and counselling | Individual information giving and skills training in self-care and sensitivity to infant. | Daily one hour for 7 days pre-discharge / 4 home visits, 7 day pre- to 90 days post-discharge. | NICU nurse trained to deliver intervention. | Maternal stress at 12 months follow-up; assessed with PSI. |
| Melnyk 2001  (18, 28) | P 26-36 & LBW | Education and counselling | Self-directed materials in infant care, development and sensitivity to infant. | 4 phase programme, 2-4 days post-birth to 1-week post-discharge. | Self-directed | Cognitive development at 4-6 months; assessed with BSID. Maternal anxiety at 1-2 months; assessed with State Trait Anxiety Inventory (STAI). Maternal depression at 6 months; assessed with Profile of Mood States. |
| Milgrom 2016  (29) | P <30 | Education and counselling | Individual information giving and skills training in self-care and sensitivity to infant. | Weekly for 9 weeks in NICU / at home. | Psychologist with experience of preterm populations. | Infant temperament at 6 months; assessed by Short Temperament Scale. |
| Moudi 2019  (30) | P 23-37 | Education and counselling | Individual information giving and skills training, with materials, in care of infant. | 4 sessions (60-90 mins each) in NICU. Researcher available for additional telephone support, 2-4 days post-birth to discharge. | Researcher. | Maternal anxiety at 1-2 months; assessed with State Trait Anxiety Inventory (STAI). |
| Newnham 2009  (19) | P <37 | Education and counselling | Individual information giving and skills training in infant care, development and sensitivity to infant. | 7 (30-60 min) sessions over final 2 weeks in NICU, 1 at home and 1 hospital visit, 2 weeks pre- to 3 months post-discharge. | Researcher. | Infant temperament at 6 months; assessed by Short Temperament Scale. Mother-infant interaction at 6 months; assessed with: Synchrony Scale. Maternal depression at 6 months; assessed with EPDS. |
| Pinelli 2001  (31) | VLBW | Education and counselling | Individual skills training, with materials, in breastfeeding. | Weekly in NICU, ‘frequently’ at home after discharge, 72h following birth until 1 year or B/F discontinued. | Independent lactation consultant. | Duration of exclusive breastfeeding. |
| Thakur 2012  (32) | LBW | Education and counselling | Individual information giving in breastfeeding. | 4 sessions (x2 per month from initiation of breastfeeding for 2 months). | Not stated. | Infant weight and length at 60 days  Breastfeeding. |
| White Traut 2013  (14) | P 29-34 | Education and counselling | Individual information giving and skills training, with materials, in infant development and sensitivity to infant. | Twice daily in NICU by mother or research nurse and continued at home by mother. Two facility sessions, two home visits and two telephone calls. Study entry (or 32 weeks) to 1-month post-discharge. | Research nurse. | Mother-infant interaction at 6 weeks; assessed with: Nursing Child Assessment Satellite Training–Feeding Scale (NCAST-Feeding). |
| Wu 2014  (33) | P &  VLBW | Education and counselling | Individual information giving and skills training in infant care and development. | 5 sessions NICU/ 8 home, within 7 days birth to 12 months CA. | Nurse & physical therapist. | Mother-infant interaction at 12 months follow-up; assessed with: Free-play procedure. |
| Zelkowitz 2009  (34) | VLBW | Education and counselling | Individual information giving, with materials, in self-care and sensitivity to infant. | 5 (1 hour sessions NICU) from average 33 days post-birth / 1 home 2-4 weeks post- discharge. | Nurse, psychologist / graduate student trained to deliver intervention. | Maternal anxiety at 6 months; assessed with STAI. |
| Mohammadian 2021  (35) | P 34-37 | Education and counselling | Individual information giving with breastfeeding, health conditions and skin to skin. | Face to face plus telephone support. 14 days of daily continuous supportive counselling by telephone after neonatal discharge. | Nurse. | Breastfeeding self-efficacy at 1, 2, 3, 4 months. |
| Korgali 2022  (36) | P 32-37 | Education and counselling | Individual information giving. | 2-hour home visit one week after discharge then month 1, 2, 3 to give information about health of infant, breastfeeding and parent-baby relationship. | Paediatrician and paediatric nurse. | Breastfeeding, depression, anxiety, infant character perception scale at 1, 3, 6, 12 months corrected age. |
| Huw 2023  (37) | P 32-37 | Education and counselling | Individual information giving, with detail and materials on massage and infant care. | One session provided by qualified practitioner and asked to perform twice daily. | Nurse and qualified lecturer. | Infant weight, parent attachment and stress at baseline at 1, 2, 4, 8 and 12 weeks. |
| Hadi 2022  (15) | LBW | Education and counselling | Individual information giving, with materials on kangaroo care, breastfeeding and handwashing. | 3 sessions on the second and sixth week. | primary health centre nurses trained in LBW infant care. | Breastfeeding, infant care practices, handwashing at 2, 6 and 12 weeks. |
| Jang 2021  (38) | P 34-36 | Education and counselling | Individual information giving, with materials on breastfeeding. | 5 sessions once a week. | Lactation consultant. | Breastfeeding at 1, 2, 3 and 4 weeks. |
| Akhbari Ziegler 2021  (39) | P <32 | Education and counselling | Individual information giving, with support from a coach at home to stimulate infant motor development. | Weekly face to face sessions 30-45 mins duration at home for 6 months. | Coach | Infant motor profile (median and range), family empowerment scale at 3, 6 months and 18 months corrected age. |
| Agrasada 2005  (40) | LBW | Home visits | Individual information giving and skills training in breastfeeding. | 8 home visits, 3 days following birth until 5.5 months. | Trained village volunteers. | Exclusive breastfeeding at 6 months. |
| Baraldi 2024  (52) | P <28 | Home visits intervention. | Individual information giving and training in infant care and interaction using a strength-based intervention, intended to consolidate the positive parent–child interplay through ten home visits plus phone calls. | One year after recruitment includes 10 visits and two telephone calls | special trained interventionist | Neurodevelopmental (Emotional availability) at 12 months corrected age |
| Gardner 2003  (41) | LBW | Home visits | Individual information giving, materials in sensitivity to infant. | Weekly 1 h home visits for 8 weeks. | Community health workers. | Bayley scales of infant development; at 10-12 months. |
| Gunn 2000  (42) | P <37 | Home visits | Individual breastfeeding support. | Daily visits for 7-10 days post-discharge. Telephone support available. | Home care nurse specialists with neonatal experience. | Number exclusively breastfeeding at 6 months. |
| Eun Sun 2020  (43) | P <37 | Home visits | Individual and group information giving and skills training in care of and sensitivity to infant. | 1 or 2 home visits per month for 6 months, plus group support sessions. | Experienced NICU nurse and a community visiting nurse. | Maternal stress at 6 months; PSI. |
| Koldewijn 2005, 2009  (44, 45) | P <32 | Home visits | Individual information giving, in care planning, infant development and sensitivity to infant. | 6-8 (1h) home visits as required. 1-week post-discharge to 6 months. | Trained paediatric physical therapists. | Infant temperament at 6 months; Infant behavioural assessment (IBA). |
| Mazumder 2019  (46) | LBW | Home visits | Individual information giving and skills training in breastfeeding and KMC. | 9 home visits (30-40mins) at 1-3, 5, 7, 10, 14, 21, 28 days. | Intensively trained intervention worker (not HW). | Infant mortality up to 180 days  Number exclusively breastfeeding at 6 months. |
| Meijessen 2010; 2011  (47, 48) | P or VLBW | Home visits | Individual information giving, in care planning, infant development and sensitivity to infant. | 6-8 (1h) home visits as required until 12 months CA. | Experienced trained paediatric physical therapist. | Maternal stress at 12 months; PSI. |
| Sinha 2021, 2022  (49, 53) | LBW | Home visits | Individual information giving and skills training in breastfeeding and KMC. | 9 home visits (30-40mins) at 1-3, 5, 7, 10, 14, 21, 28 days. | Intensively trained intervention worker (not HW). | Maternal depression at 28 days; Patient health questionnaire. |
| Taneja 2020  (50) | LBW | Home visits | Individual information giving and skills training in breastfeeding and KMC. | 9 home visits (30-40mins) at 1-3, 5, 7, 10, 14, 21, 28 days. | Intensively trained intervention worker (not HW). | Cognitive development. Bayley scales of infant development; at 10-12 months. |
| Youn 2021  (51) | P <30 or VLBW | Home visits | Individual information giving and group skills training in infant care, development and sensitivity to infant. | 4 home visits (Discharge until 2 months CA) 12 x 90 min group sessions with physiotherapist. | Specialist nurse and physiotherapist (infant neurodevelopment). | Cognitive and motor development at 10 months. Bayley scales of infant development. Mother/infant attachment at 6 months; Mother-Child Attachment (MCA) scale. |
| Ingram 2016  (54) | P 27-<34 | Discharge preparedness | Planning for early discharge. Care of infant. | As required, for 5 weeks, between 27-33 weeks. | NICU staff. | Emergency hospital visits; number until 2 months. |
| Lee 2019  (55) | P <32 | Discharge preparedness | Planning for discharge. Care of infant. | 3 sessions and 1 FU tele call. 34 weeks to 72h post-discharge. | Neonatal nurse consultant. | Maternal stress 1-2 months; assessed with Perceived Stress Scale (C-PSS). |
| Neyestani 2017  (56) | P 30-35 | Discharge preparedness | Planning for discharge. Care of infant.  Materials. Included parent-directed contact following discharge if required. | 4 (35-40 min) sessions plus 4 telephone contacts (x1 per week, 5-10 mins) after discharge. 48h from birth until 4wks post-discharge. | Nurse. | Maternal quality of life at 4 weeks; assessed with WHOQOL-BREF. |
| Ortenstrand 2001  (57) | P <37 | Discharge preparedness | Planning for discharge and early discharge. Care of infant. | Care planning session and domiciliary care visits (number unclear). Pre-discharge until domiciliary care completed. | Project nurse (neonatal trained). | Maternal anxiety at 3 months; assessed with STAI. |
| Ericson 2018  (58) | P <37 | Digital communication | Individual support/communication in breastfeeding. | Daily telephone call from discharge for 14 days. | Breastfeeding support team (NICU staff trained for 2 days). | Exclusive breastfeeding at 1-2 months. |
| Hagi-Pederson 2020  (59) | P <37 | Digital communication | Individual support/communication in breastfeeding. | 2-3 consultations per week by video following introduction in NICU, until discharge programme completion. | Nurse trained in early in-home care and trained in smartphone application, video consultations. | Exclusive breastfeeding at 1-2 months. Maternal-infant interaction at 1 month; assessed with: Mother and Baby Interaction Scale (MABISC). |
| Luu 2017  (60) | P <30 | Digital communication | Individual support/communication, with materials, in infant care. | 3x in-person workshop and 4 web-based modules (commenced in NICU), until 12 months. | Certified occupational therapist trained in developmental care. | Maternal-infant interaction at 4 months; assessed with: Parental Cognitions and Conduct Toward the Infant Scale. |
| Robinson 2016  (61) | P 27-37 | Digital communication | Individual support/communication in infant care. | 3x Skype calls per week with messaging option, to discharge from home health care. | developmental care. | Emergency hospital visits up to 2 months post-discharge. |
| Bahmanpour 2023  (62) | P 28-36 | Digital communication | Individual support and communication with materials in infant care. | Daily education materials for 4 weeks. Social media following with educational files via mobile phone on care provision for newborn. Personal contact with researchers to review materials and answer questions. | Nurse. | Feelings of ‘hope’ as per the Hope Scale (mean and SD) and maternal perceived self-efficacy (PMPS-Q mean and SD) before the intervention, immediately after and at 4 weeks. |
| Yan 2022  (63) | P (no definition) | Digital intervention. | Health education app on the care and feeding of premature infants and the recognition and treatment of complications plus online chat to address parents' problems |  | Self-directed | Anxiety and depression at one month of age. Self-Rating Anxiety Scale; SDS, Self-Rating Depression Scale |
| Zhang 2023  (64) | P 28 – 36+6 | Digital intervention. | Online classes, online counselling, 5 lessons; for main caregivers via Rain Classroom, a WeChat-imbedded software | 8 hours across 3 months | Self-directed | Infant Developmental Scores  Ages & Stages Questionnaires at one month and 3 months |
| Neila-Vilen  2016  (65) | P <35 | Peer support | Individual support and materials in breastfeeding. | As required (facility / home), to 12 months. | Volunteers with experience of B/F preterm infants (no training given).  Midwife available to answer B/F questions. | Duration of exclusive breastfeeding |
