# Supplementary figures and images for: Interventions to support parents, families and caregivers in caring for preterm or low birth weight infants at home: A systematic review and meta-analysis
Source: PLOS Glob Public Health. 2026 Feb 10;6(2):e0005690. doi: 10.1371/journal.pgph.0005690 (PMC12890145; doi:10.1371/journal.pgph.0005690)

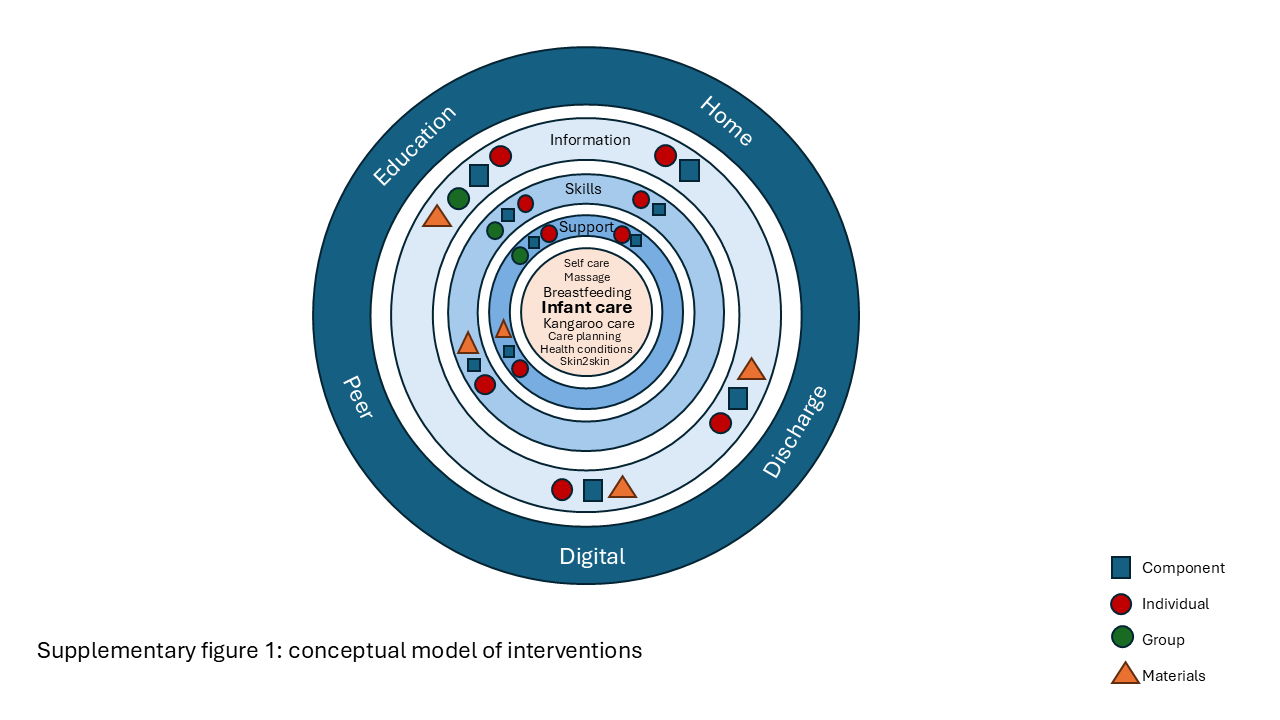

Supplement: S1 Fig — (TIF) [file pgph.0005690.s008.tif]

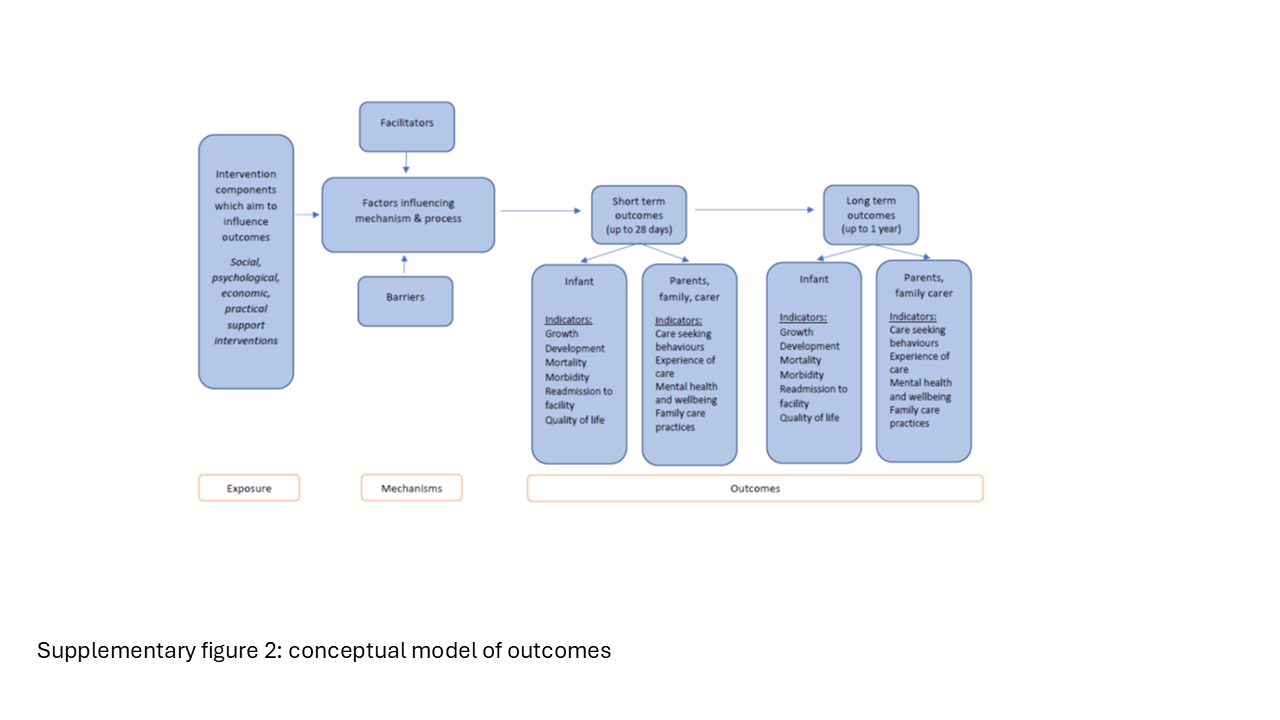

Supplement: S2 Fig — (TIF) [file pgph.0005690.s009.tif]

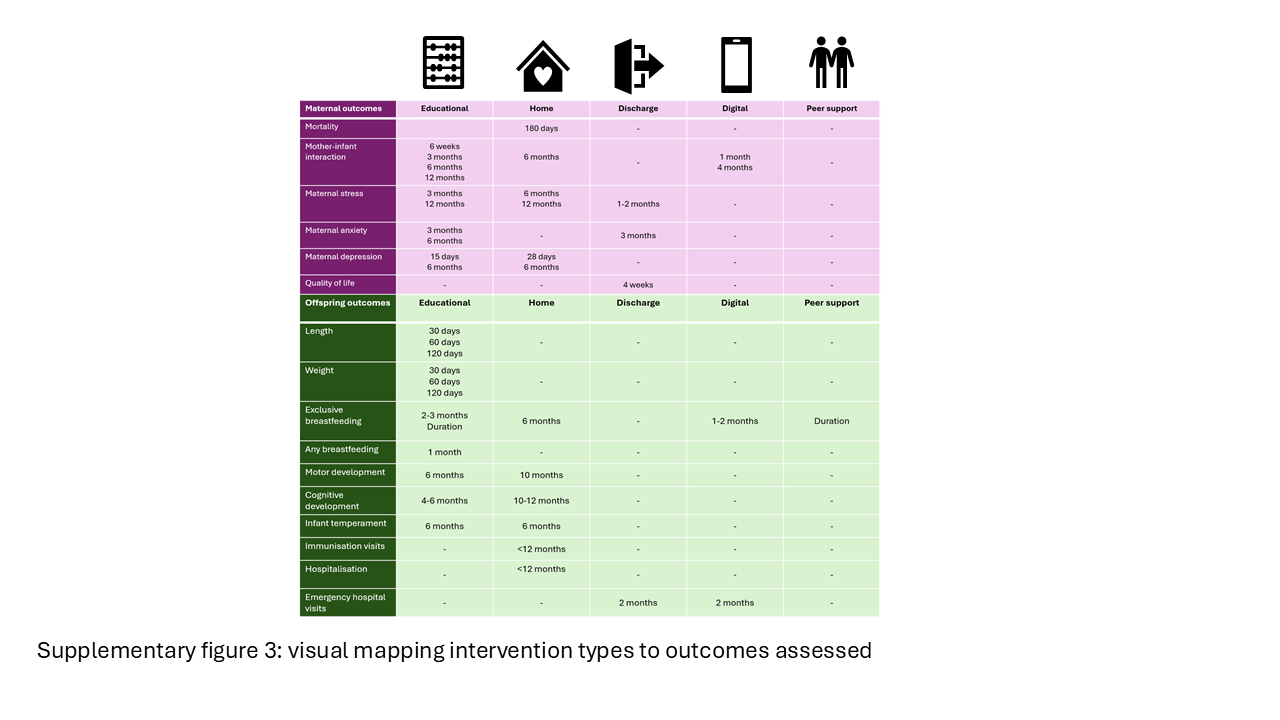

Supplement: S3 Fig — (TIF) [file pgph.0005690.s010.tif]
